# Sex‑specific cardiovascular risk in estrogen‑treated androgen‑deprived males: metabolic characterization of glucose, adipose, and lipid pathways

**DOI:** 10.1186/s12933-025-03059-y

**Published:** 2026-02-02

**Authors:** Ariel S. Thorson, Kelsey Pinckard Schaefers, Bridget Litts, Jeffrey Rein, Sharmila Adapa, Sivaprakasam Chinnarasu, Kathryn Shapiro, Yutian Zhao, Sophia Yu, Blake Recupido, Kyla J. Streng, In Sook Ahn, Ruirui Lan, Olivia Pierre-Louis, Drucilla Forson, Marcus Bennett, Hannah Luviano, Mohammad Saleem, Lin Zhu, Xia Yang, Annet Kirabo, John M Stafford

**Affiliations:** 1 Department of Medicine, Division of Endocrinology, Diabetes, and Metabolism, The Ohio State University, Wexner Medical Center, OH Colombus, USA; 2 Department of Molecular Physiology and Biophysics, Vanderbilt University, Nashville, TN USA; 3 Department of Medicine, Division of Diabetes, Endocrinology and Metabolism, Vanderbilt University Medical Center, Nashville, TN USA; 4 Department of Medicine, Division of Genetic Medicine and Clinical Pharmacology, Vanderbilt University Medical Center, Nashville, TN USA; 5 Tennessee Valley Health System, Veterans Affairs, Nashville, TN USA; 6 Department of Integrative Biology and Physiology, University of California, Los Angeles, , CA USA

## Abstract

**Background:**

Estrogen therapy and androgen‑deprivation were once combined to treat prostate cancer (PrCa). Clinical studies later showed that prolonged estrogen exposure in androgen‑deprived men raises cardiovascular disease (CVD) risk, yet the metabolic pathways responsible remain unclear.

**Methods:**

We generated an androgen‑deprived, 17β‑estradiol (E2)–treated mouse model by gonadectomizing male C57BL/6 J mice and implanting sub‑cutaneous delayed‑release E2 or vehicle pellets. Mice received a Western‑style diet and were housed at thermoneutrality to accelerate CVD‑risk phenotypes. Metabolic profiling included hyperinsulinemic‑euglycemic clamps, oral lipid and pyruvate tolerance tests, flow cytometry of immune cells, and single‑nucleus RNA sequencing of liver tissue.

**Results:**

In hypogonadal males, E2 treatment induced several metabolic disturbances. During clamps, E2‑treated mice showed markedly elevated gluconeogenesis, corroborated by higher glucose peaks and AUC during pyruvate tolerance testing and by up‑regulation of hepatic Pck1 mRNA. Triglyceride (TG) clearance, which improves with E2 in females, was impaired in E2‑treated males: oral lipid‑tolerance testing revealed prolonged TG excursions, reduced maximal lipase activity, lower non‑lipase clearance at 6 h post-OLTT, and decreased free‑fatty‑acid peak levels. Hepatic lipase, VLDL clearance receptors Ldlr and Lrp1, and microsomal triglyceride transfer protein (MTP) transcripts were down‑regulated. SnRNA‑seq showed suppression of lipid‑clearance genes with E2 treatment in males. Subcutaneous adipocytes were hypertrophic, and flow cytometry identified increased TNFα‑positive macrophages, an inflammatory milieu that could promote insulin resistance. Cardiac morphology was modestly altered; E2‑treated males exhibited a larger left‑ventricular end‑diastolic diameter, while ejection fraction and arterial pressure remained unchanged.

**Conclusion:**

Estradiol administration in androgen‑deprived male mice produces a constellation of metabolic derangements—including enhanced hepatic gluconeogenesis, impaired TG clearance, and inflammatory adipocyte hypertrophy—that likely underlie the increased CVD risk observed clinically. The identified molecular nodes (PEPCK, hepatic lipase, LRP1, LDLR, MTP, and adipose‑macrophage TNFα) provide potential targets for mitigating estrogen‑induced CVD risk while preserving its therapeutic benefit for PrCa.

**Graphical abstract:**

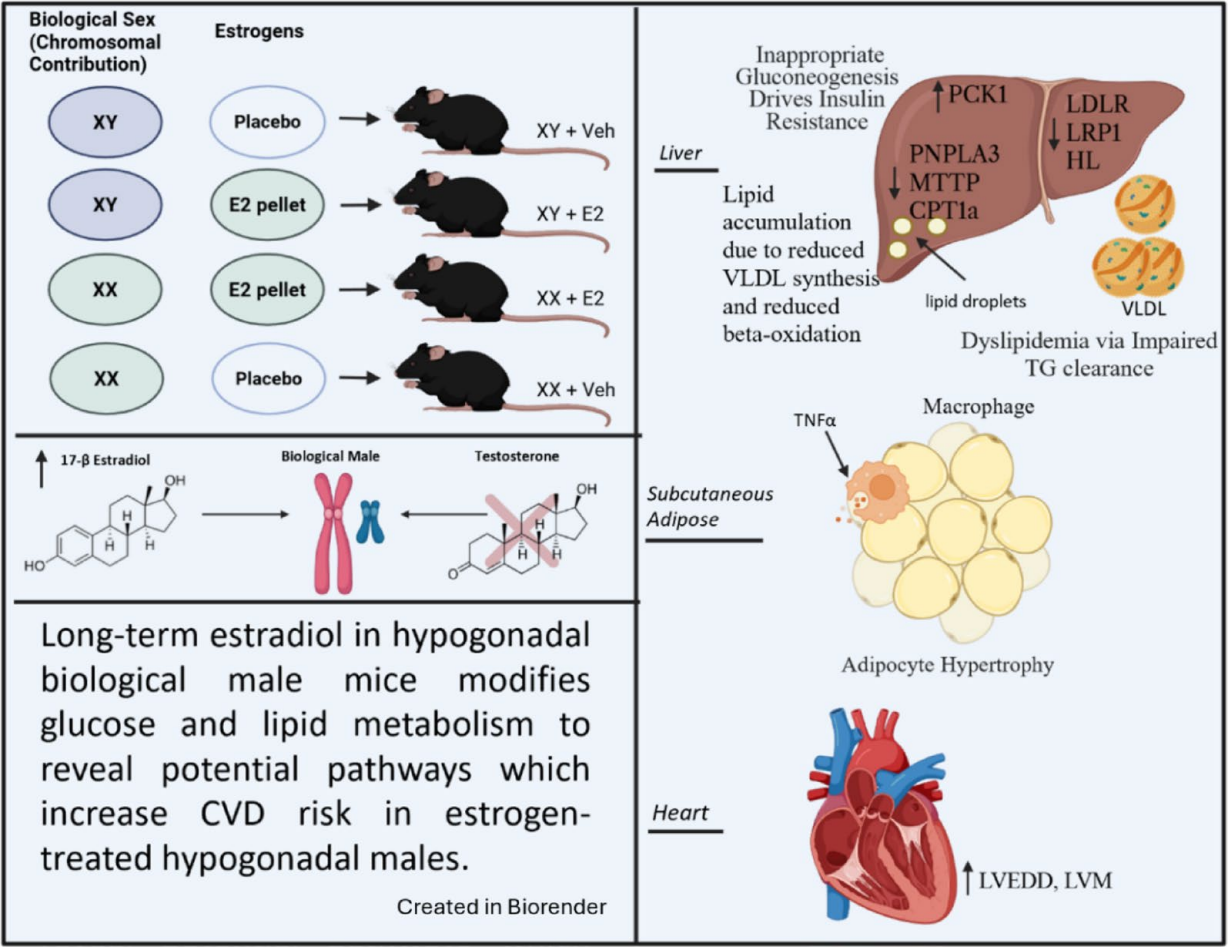

**Supplementary Information:**

The online version contains supplementary material available at 10.1186/s12933-025-03059-y.

## Introduction

Hypogonadism is defined by clinically low testosterone in men [1]. Clinically hypogonadal men taking estrogens may be at increased risk of cardiovascular disease (CVD) compared with men in the general population [1–3]. The risk associated with low testosterone plus high estrogen in men is higher than the elevated CVD risk due to low testosterone alone [3–6]. The additive risk of estrogens in hypogonadal males is paradoxical, because estrogens are cardioprotective and reduce CVD risk in cis‑gender premenopausal women [4].

Estrogenic benefits in reducing CVD risk in women are partly due to beneficial metabolic pathway modifications, but it is not well understood how these CVD‑risk‑modifying pathways are altered in hypogonadal men [7–9]. 17 β-Estradiol (E2), the predominant circulating estrogen in women, increases the secretion and clearance of very‑low‑density lipoprotein (VLDL) and augments high‑density lipoprotein (HDL) concentrations and function [10–12]. Estrogens also reduce the risk for insulin resistance and metabolic‑associated fatty‑liver disease (MAFLD), two independent risk factors for CVD [13, 14]. This process is partly mediated by estrogenic binding to liver estrogen‑receptor‑α (ERα), leading to beneficial liver effects including suppression of hepatic glucose production, increased lipid clearance, and improved insulin action [13–15]. In females, estrogens also modify adipocyte uptake of fats such that excess lipids are preferentially deposited in subcutaneous depots rather than visceral depots [17]. This shift away from abdominal adipose tissue is associated with a reduced risk of CVD [18]. To accommodate the increased lipid load in subcutaneous fat, estrogens also promote adipocyte hyperplasia—a morphology linked to reduced adipose‑associated inflammation and cellular dysfunction [17, 19–21]. These estrogenic pathways may underlie the cumulative CVD‑risk‑reduction observed in premenopausal women. The increased CVD mortality observed in hypogonadal, estrogen‑treated men suggests discordance in estrogenic modification of these pathways could contribute to estrogenic harm in the hypogonadal male population, but the pathways responsible remain poorly understood. Because the cardioprotective actions of estrogens in females are largely mediated through ERα binding to estrogen‑response elements (EREs) in gene promoter regions, we included a female‑E2 control group to determine whether known genomic pathways reducing CVD risk in females operate similarly, or become discordantly regulated, in hypogonadal males [4, 16]. Comparing males and females receiving identical E2 exposure allows us to isolate sex‑specific and sex-independent molecular mechanisms. Identifying CVD risk-modifying pathway divergence points could reveal where estrogens confer benefit in women but harm in hypogonadal men.

Estrogens were once aneffective therapy for the treatment of prostate cancer (PrCa) in men. Metastatic and high-risk non-metastatic PrCa is treated with androgen deprivation to induce hypogonadism, as the majority of PrCa is androgen‑sensitive [22]. Diethylstilbestrol (DES) was utilized as an adjuvant therapy for PrCa treatment in the United States from the 1940s through the late 1980s [23–25]. Patients treated with 1–5 mg of DES and androgen‑deprivation via orchidectomy reported significantly improved quality of life, an 85% reduction in death from stage III prostate cancer, and a 74% reduction in death from stage IV prostate cancer compared with patients who received orchidectomy alone [24]. DES was more effective at prolonging survival and reducing cachexia in patients with metastatic PrCa when compared with luteinizing‑hormone‑releasing hormone (LHRH) agonists, the current standard of care for androgen deprivation [26]. However, FDA approval for DES was withdrawn by the drug maker in 2000 in response to accumulating evidence of cardiotoxicity, marking the end of estrogens in the treatment of PrCa [11].

Identifying the pathways through which estrogens mediate harmful CVD effects in hypogonadal men could also reduce mortality risk in an estimated 720,000 individuals across the US and EU who are taking estrogens and anti‑androgens [8, 9]. E2 is currently used in conjunction with anti‑androgens to treat gender dysphoria and has been shown to reduce risk of suicide, improve quality of life, and alleviate severe depression [10, 22]. E2 treatment has been shown to increase CVD risk and mortality in this population by as much as threefold compared with men in the general population [2, 23, 27]. Importantly, E2 has a more favorable coagulation profile than DES. Adjusted VTE incidence with E2 is either unchanged or only modestly increased (≈ 1.2‑fold), whereas DES historically raised VTE rates 3‑ to 5‑fold [2, 4–6]. This discrepancy suggests that mechanisms other than hypercoagulability drive the excess CVD risk observed in hypogonadal, estrogen‑treated males.

Estrogenic treatment in androgen‑deprived PrCa patients is known to improve quality of life, decrease PrCa progression, and reduce death from PrCa; however, increased CVD mortality prevents patients from accessing this treatment [3, 25]. Broad re‑introduction of estrogens for PrCa treatment will only be feasible once the cause of increased CVD harm is understood, making the study of estrogenic changes to CVD risk factors in hypogonadal males a critical area of research. To the best of our knowledge, this is the first paper to comprehensively examine CVD risk through the study of glucose metabolism, liver histological changes, lipid metabolism, and adipose morphology in a hypogonadal male system administered long‑term E2. Here, we examine the discordant and beneficial effects of E2 in androgen‑deprived males versus biological females and androgen‑deprived placebo males.

## Results

### Altered left ventricular morphology despite favorable body composition changes in estrogen-treated androgen-deprived male mice

**Fig. 1:**
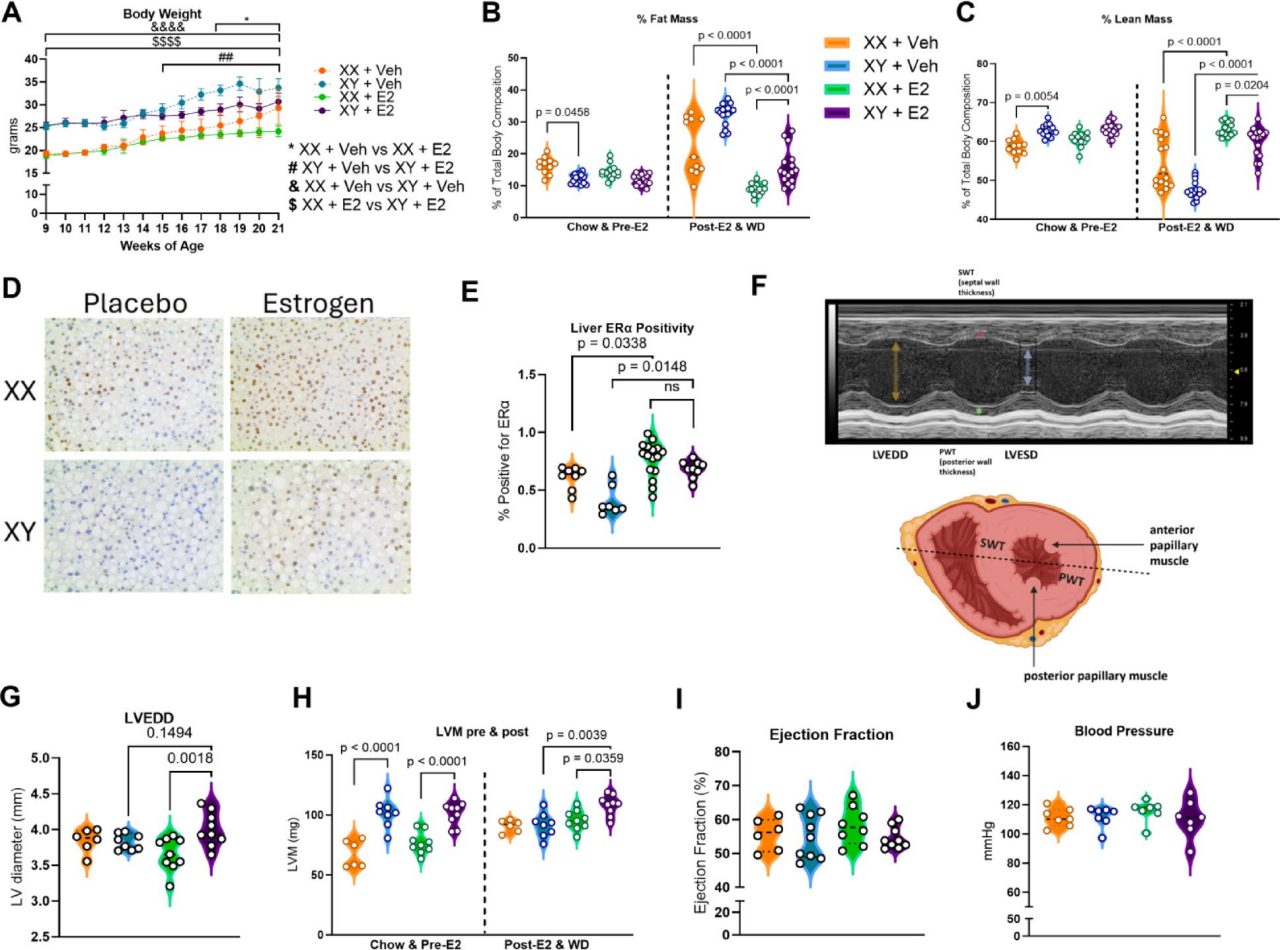
Abnormal Cardiac Morphology Despite Favorable Changes to Body Composition in E2-Treated, Androgen-Deprived Males. **A** Body weight decreased for XY mice following gonadectomy and estrogen pellet placement at 11 weeks. n = 11–16; Two-way ANOVA with Tukey’s Multiple Comparisons Test. **B** Percent fat mass of the estrogen-treated mice decreased significantly in a sex-independent manner following 10-week E2 treatment. n = 11–18; One-way ANOVA with Bonferroni’s Multiple Comparison Test. **C** NMR showed increased lean mass as a percent of body composition in E2-treated males versus placebo males at study termination. n = 11–18; Two-way ANOVA with Tukey’s Multiple Comparison Test. **D** ERα immunohistochemical staining in liver samples at study termination reveals increased positivity in estrogen-treated groups. ERα antibody stain is brown, and nuclear stain is blue. **E** Analysis of hepatocyte staining for ERα positivity shows significant increase in ERα + cells in groups with estrogen exposure, regardless of biological sex. n = 7–16; Kruskal–Wallis Test with Dunn’s Test for Multiple Comparisons. **F** Echocardiography image of left ventricle in short axis with heart diagram illustrating transducer positioning. **H G** LVEDD is increased in estrogen-treated males versus females. n = 6–9; One-way ANOVA with Bonferroni’s Multiple Comparison Test. **H** Short-axis echocardiography demonstrated increased LV mass in estrogen-treated males vs placebo males. n = 6–9; Two-way ANOVA with Tukey’s Multiple Comparison Test. **I** EF is unchanged across treatment groups. n = 6–9; One-way ANOVA with Bonferroni’s Multiple Comparison Test. **J** Blood pressure via tail cuff measurement is unchanged between groups. n = 7; Kruskal–Wallis Test with Dunn’s Test for Multiple Comparisons

To determine if beneficial estrogenic changes to body weight and composition were conserved in males, body weight was recorded weekly and nuclear magnetic resonance (NMR) imaging was used to quantify fat and lean mass compositional changes. E2 treatment slowed weight gain in both biological males and females despite exposure to obesogenic western-diet and thermoneutral conditions (Fig. 1A). Our results showed a reduced percent fat mass in E2-treated androgen-deprived males versus placebo males at study termination (*p* < 0.0001) (Fig. 1B). Lean mass at study termination was significantly higher in E2-treated males than placebo males (*p* < 0.0001) (Fig. 1C).

To confirm that liver ERα, an estrogenic pathway target identified as partly mediating CVD protection in females and a potential regulator of E2’s effects within males, was upregulated in the liver of E2-treated males, liver tissue was immunohistochemically stained for ERα (Fig. 1D). A significantly greater proportion of hepatocytes stained positive for ERα in the E2-treated cohorts than in the placebo cohorts, indicating that E2 treatment led to an increase in estrogen-responsive hepatocytes in male mice (*p* = 0.0222) and in female mice (*p* = 0.0338) (Fig. 1E).

Echocardiography was performed to examine whether E2 treatment in hypogonadal males led to adverse cardiac structural and functional changes (Fig. 1F). Left ventricular end diastolic diameter (LVEDD) at study conclusion was found to be significantly increased in the E2-treated males versus the E2-treated females (*p* = 0.0018) (Fig. 1G). LVEDD trended toward being higher in the E2-treated males than placebo males, but this did not reach the level of significance (*p* = 0.1494). Left ventricular mass (LVM) in the estrogen-treated hypogonadal males exceeded that of the placebo male group (*p* = 0.0039) (Fig. 1H). Ejection fraction (EF) was calculated to determine whether increased LVEDD reduced left ventricular contractile capacity in E2-treated males, but there was no significant change to EF at study termination (Fig. 1I). LVM elevation in the setting of preserved EF necessitated a study of blood pressure (BP) dynamics. BP via tail vein cuff measurement revealed no changes to BP between groups, indicating that the abnormal cardiac morphology observed in the E2-treated, androgen-deprived males was not due to elevated BP (Fig. 1J).

### Estradiol administration significantly improved glucose tolerance in E2-treated androgen-deprived males but increased age accumulation

**Fig. 2:**
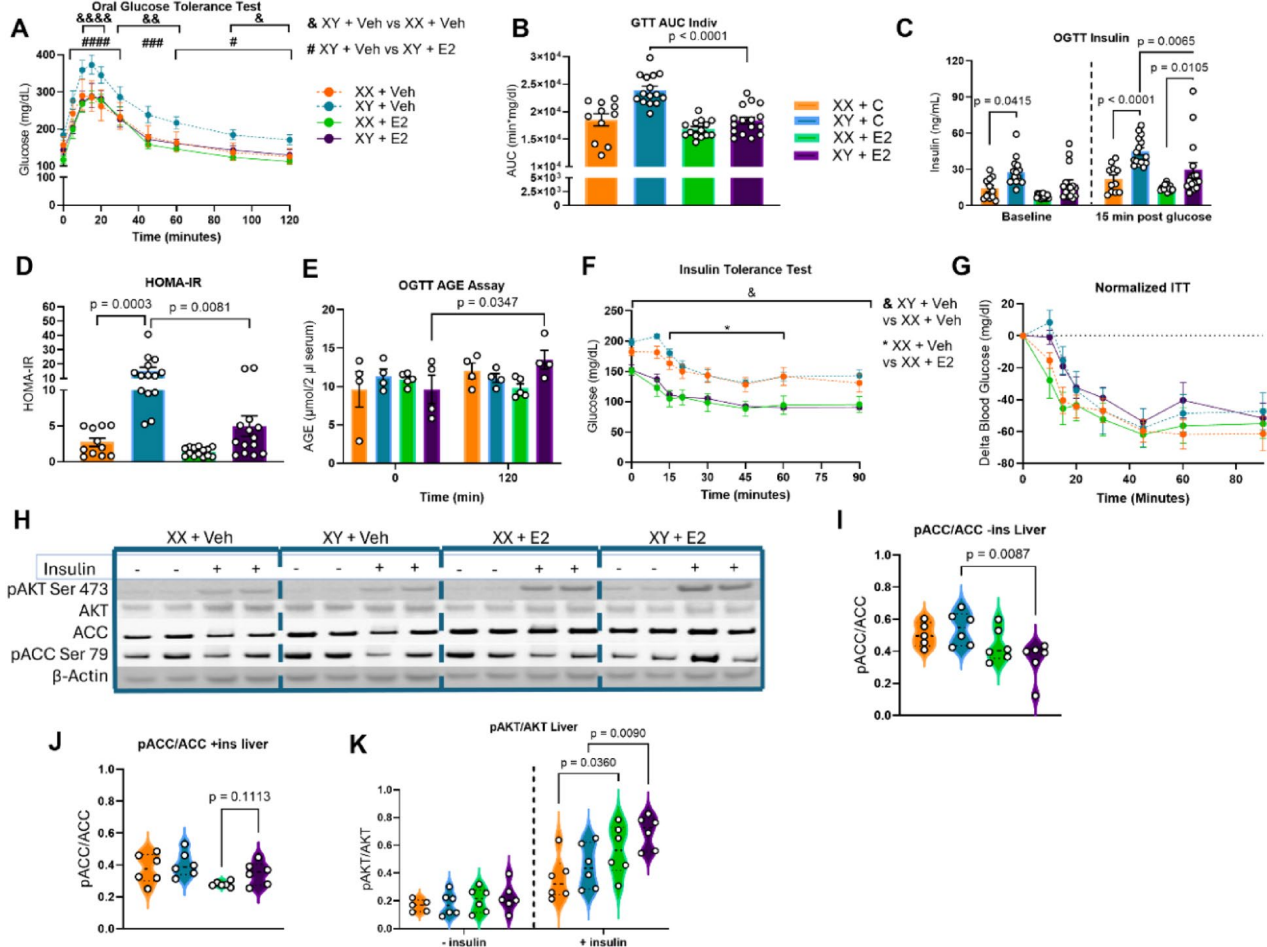
Intermediate glucose benefit to XY + E2 mice relative to XY + Veh group. **A** Oral glucose tolerance test (OGTT), measuring glucose levels following oral glucose gavage of 2 g/kg lean mass of 20% dextrose in five-hour fasted mice shows significantly lower glucose levels in placebo males versus E2-treated males. n = 11–16; Two-way ANOVA with Tukey’s Multiple Comparisons Test. *p < 0.05, **p < 0.01, ***p < 0.001, ****p < 0.0001. **B** OGTT area under the curve (AUC) shows reduction in E2-treated males versus placebo males. n = 7–16; One-way ANOVA with Bonferroni’s Multiple Comparison Test. **C** Circulating insulin levels at baseline and at 15 min post-gavage. n = 7–16; Two-way ANOVA with Tukey’s Multiple Comparisons Test. **D** Homeostatic measure for the assessment of insulin resistance (HOMA-IR). HOMA-IR at 20 weeks was reduced in E2-treated males versus placebo males. n = 11–14; Kruskal–Wallis Test with Dunn’s Test for Multiple Comparisons. **E** Advanced glycation end-product (AGE) assay from tail vein blood at baseline and at 120 min post-OGTT reveals increased AGE at 120 min post-OGTT in E2-treated males. n = 4–5; Two-way ANOVA with Tukey’s Multiple Comparisons Test. **F** Insulin tolerance test (ITT) via intraperitoneal insulin injection of 1 UI/kg lean mass. n = 5–7, Two-way ANOVA with Tukey’s Multiple Comparisons Test. **G** Normalization of ITT to baseline glucose. **I** Liver western blot protein quantification in ± insulin conditions. **I** Densitometry analysis of Western Blot for pACC Ser^79^ to total ACC under—insulin conditions reveals significant decrease in E2-treated males versus placebo males. n = 5,6; Kruskal–Wallis Test with Dunn’s Test. **J** pACC Ser^79^ to total ACC densitometry western blot analysis under + insulin conditions reveal trend toward increase in E2-treated males versus females. n = 6; Kruskal–Wallis Test with Dunn’s Test. **K** pAKT Ser^473^ to total AKT densitometry analysis of western blot data show increase in the estrogen-treated groups relative to placebos. n = 5,6; Two-way ANOVA with Tukey’s Multiple Comparisons Test

To assess how E2 treament influences glycemic control in the hypogonadal male, we performed oral glucose tolerance testing (OGTT). E2‑treated males showed a significant reduction in blood glucose from 5 min after gavage through 120 min compared with placebo males (Fig. 2A). In contrast, placebo males maintained higher glucose levels than placebo females throughout the same interval. Overall glucose excursion was significantly lower in the E2-treated males than the placebo males (Fig. 2B). Fasting insulin levels were significantly lower in the placebo females than in the placebo males, but E2 treatment had no effect on baseline insulin levels (Fig. 2C). Together, these data suggest a sex-independent benefit of E2 in improving glucose tolerance.

Further examination of post-gavage insulin levels during the OGTT, however, suggested that improved glucose control in E2-treated males may be due to higher post-prandial insulin levels, rather than due to improved insulin sensitivity. Insulin levels at 15 min post-gavage were significantly elevated in the E2-treated males versus the E2-treated females, despite all mice being dosed with glucose per kilogram of lean mass to control for body weight and body compositional differences (Fig. 2C). This presents a conflicting picture, wherein E2‑treated males required more insulin to achieve glucose excursions comparable to those of E2‑treated females, but E2-treated males did not exhibit fasting insulin resistance via HOMA-IR (Fig. 2D). To the contrary, E2 treatment significantly decreased HOMA-IR in hypogonadal placebo males versus both placebo females and E2-treated males (Fig. 2D).

The final analysis of the OGTT probed for inflammatory, reactive species formation. Glucose can non-enzymatically adduct to proteins and lipids irreversibly to form reactive and pro-inflammatory compounds known as advanced glycation end products (AGEs). To test for changes to AGE levels in serum following a glucose challenge, serum was collected in the mice at baseline and at 120 min post-OGTT. AGE levels rose only in E2-treated hypogonadal males following glucose administration (Fig. 2E), suggesting that pro-inflammatory carbohydrate byproduct formation may be augmented in E2-treated males.

To examine potential insulin resistance further, an insulin tolerance test (ITT) was performed, with dosing according to lean mass. The ITT initially revealed estrogen-dependent differences in glucose lowering in response to insulin challenge; placebo groups exhibited blunted insulin response, relative to sex-matched E2-treated groups (Fig. 2F). However, when the ITT was normalized to baseline glucose, the estrogenic differences were eliminated, indicating that higher fasting glucose levels in the placebo groups, rather than E2 treatment benefiting insulin action in the E2-treated groups, were responsible for observed differences in the ITT (Fig. 2G).

Quantification of intracellular protein levels was performed in + and – insulin conditions for liver tissue (Fig. 2H). Phosphorylation of acetyl‑CoA carboxylase (ACC) at Ser⁷⁹ was significantly reduced in E2‑treated males versus placebo males under fasting conditions (Fig. 2I). This could indicate an impairment in adenosine monophosphate kinase (AMPK) phosphorylation of ACC or an increase in its dephosphorylation. Liver phosphorylation of ACC following insulin injection was not different between E2-treated males and placebo males, but pACC did trend higher in E2-treated males compared to E2-treated females (Fig. 2J). Because ACC phosphorylation is normally suppressed by insulin, these findings suggest that E2 does not restore appropriate ACC regulation in males when insulin is present. To gauge the liver insulin signaling cascade, protein kinase B (AKT) phosphorylation was also quantified. Insulin-mediated AKT phosphorylation at Ser^473^ was significantly increased in intensity for the E2-treated groups compared to their placebo controls (Fig. 2K). This suggests that E2 treatment, at least to the level of AKT, promotes a robust hepatic molecular response to insulin in a sex-independent manner.

## Hyperinsulinemic-euglycemic clamp reveals sex-dependent insulin resistance in males, with E2-treated males exhibiting inappropriate gluconeogenic activity and potential metabolic inflexibility

**Fig. 3:**
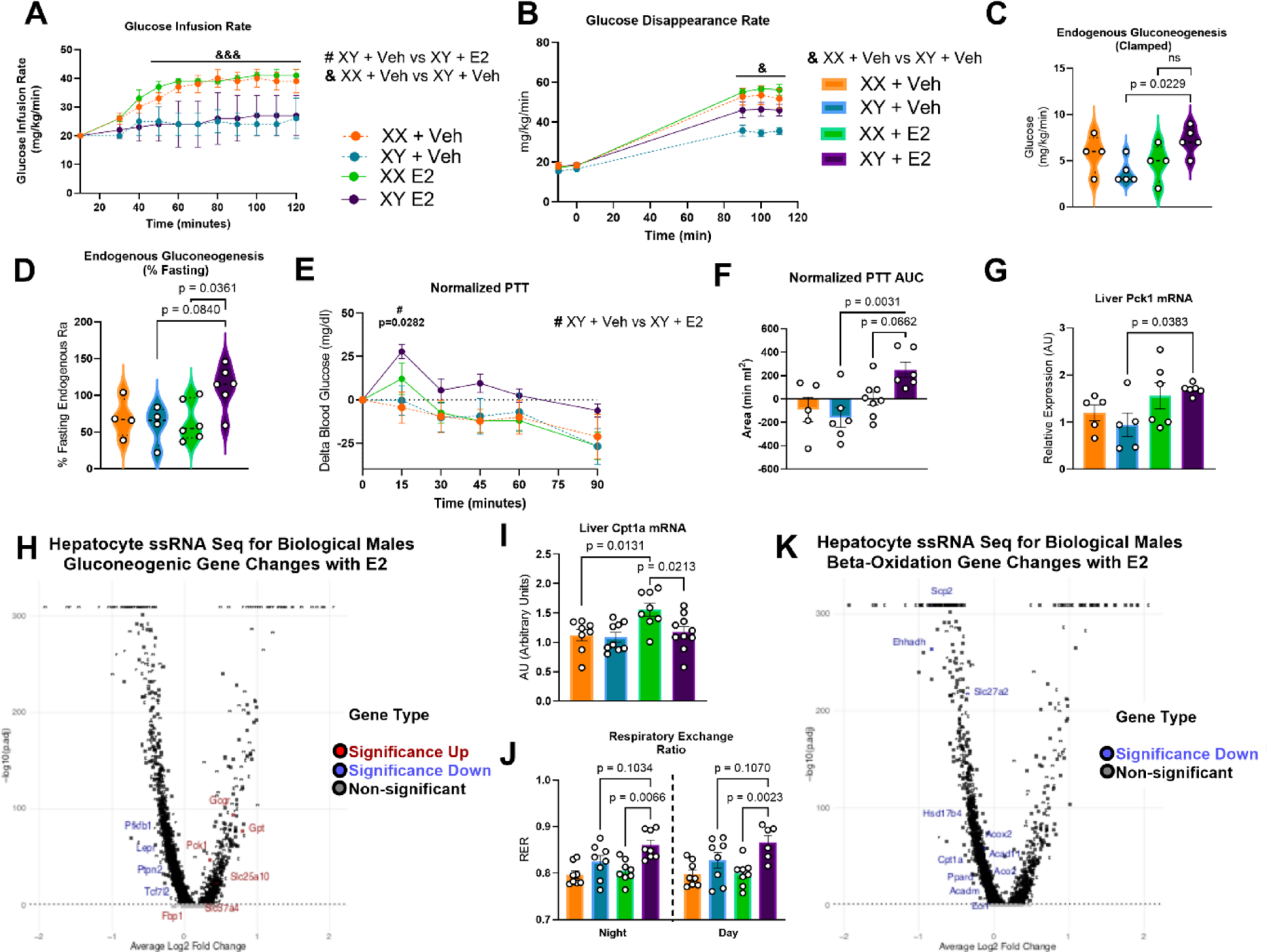
Hyperinsulinemic-Euglycemic Clamp Suggests Baseline Insulin Resistance in Androgen-Deprived E2 Treated Males is Biological Sex Dependent. **A** Hyperinsulinemic euglycemic clamp glucose infusion rates (GIR) separated by biological sex rather than E2 treatment status. n = 4–6; Two-way ANOVA with Tukey’s Multiple Comparisons Test. **B** Glucose disappearance rates over the clamp calculated by measuring disappearance of D2-glucose. n = 4–6; Two-way ANOVA with Tukey’s Multiple Comparisons Test. **C** Endogenous gluconeogenesis during the clamp calculated by measuring D2-glucose to untagged glucose shows significantly higher clamped gluconeogenesis in E2-treated males versus placebo males. n = 4–6; Kruskal–Wallis Test with Dunn’s Test. **D** Gluconeogenesis rates normalized to baseline, elevation in E2-treated males versus females. n = 4–6; Kruskal–Wallis Test with Dunn’s Test. **E** A pyruvate tolerance test (PTT) was performed via an oral gavage of 2 g/kg body weight sodium pyruvate. Normalized to baseline glucose. n = 5–8, Two-way ANOVA with Tukey’s Multiple Comparisons Test. **F** PTT area under the curve showed that E2-treated males had significantly higher AUC versus placebo males. n = 5–8, One-way ANOVA with Bonferroni’s Multiple Comparison Test. G) Reduced Pck1 liver mRNA expression in E2-treated males versus placebo males. n = 5–6; One-way ANOVA with Bonferroni’s Multiple Comparison Test. **H** Hepatocyte snRNA sequencing for gluconeogenic pathway genes in biological males, with upregulation signifying fold change increase with E2 treatment. Significantly downregulated: *Pfkfb1, Lepr, Ptpn2, Tcf7l2.* Significantly upregulated: *Gcgr, Pck1, Slc25a10, Alc37a4, Fbp1.* n = 3, **I** Cpt1a liver mRNA expression with E2 treatment in females but not in males. **J**. Indirect calorimetry data was collected at 5-min intervals. RER was averaged during each cycle (day/night). E2-treated males exhibited higher RER than E2-treated females. n = 6–8; Kruskal–Wallis Test with Dunn’s Test for Multiple Comparisons. **K** Hepatocyte snRNA sequencing for gluconeogenic pathway genes in biological males. n = 3, Multiple Testing with Bonferroni’s Multiple Comparisons Test. Significantly downregulated: *Hsd7b4, Acox1, Acox2, Aco2, Cpt1a, Ppard, Acadm, Ehhadh, Slc27a2, Scp2, Eci1*

Increased insulin levels in the E2-treated males during the OGTT indicated that baseline insulin resistance could be present in this group, but the results of the normalized ITT were inconclusive. To test this hypothesis further, a hyperinsulinemic-euglycemic clamp was performed. The clamp revealed a biological sex-dependent difference in glucose infusion rate (GIR), where biological males tolerated significantly lower GIR than females (Fig. 3A). Glucose disappearance rates of placebo males were significantly lower than the placebo females (Fig. 3B). Glucose disappearance rates in E2-treated males, however, was intermediate despite similar insulin resistance to placebo males via GIR.

One possible explanation for this difference was identified during the clamp. Endogenous clamped gluconeogenesis was significantly increased in E2-treated hypogonadal males versus placebo males, indicating an effect of E2 treatment in opposing insulin-mediated gluconeogenic suppression (p = 0.0229) (Fig. 3C). When clamped gluconeogenesis was normalized as a percent of fasting gluconeogenesis, rates in E2-treated males were significantly higher than E2-treated females (*p* = 0.0361), with a trend toward higher rates in E2-treated males than in placebo males (p = 0.0840) (Fig. 3D). Gluconeogenesis as a percent of fasting was increased with E2 treatment in males but not in females, with E2-treated males performing higher levels of gluconeogenesis during clamped conditions when normalized to fasting rates. This suggests that E2 treatment effects on augmenting gluconeogenesis during hyperinsulinemic conditions were unique to the interaction of E2 with the hypogonadal biological male system.

To further assess gluconeogenic capacity in androgen-deprived, E2-treated males, a pyruvate tolerance test (PTT) was performed. E2-treated males exhibited higher peak glucose levels during the PTT compared to placebo males (*p* = 0.0282) (Fig. 3E). The AUC from the PTT was also significantly higher in the E2-treated males than in the placebo males (*p* = 0.0031) (Fig. 3F). There was a trend toward increased excursion in E2-treated males compared with E2-treated females (*p* = 0.0662). This suggests that E2 treatment augments gluconeogenic capacity in androgen-deprived males but not in females. Indeed, liver mRNA expression for phosphoenolpyruvate carboxykinase 1 (PCK1), the rate-limiting step in gluconeogenesis, is significantly increased in E2-treated males versus placebo males (*p* = 0.0383) (Fig. 3G). Liver single nucleus RNA sequencing (snRNA seq) data confirmed upregulated gene expression of Pck1 in E2-treated males versus placebo males (Fig. 3H). This reveals a possible molecular pathway mediator of augmented gluconeogenic capacity in E2-treated males. Likewise, snRNA seq revealed upregulated gene expression of Gpt (alanine aminotransferase, providing pyruvate substrate) and Gcgr (glucagon receptor). Conversely, Pfkfb1, which regulates fructose‑2,6‑bisphosphate (an allosteric inhibitor of gluconeogenesis), was down‑regulated, and three genes that normally suppress gluconeogenesis—Lepr, Tcf7l2, and Ptpn2—showed decreased gene expression with E2 treatment in males. Together, these genes provide insight into potential molecular pathways through which gluconeogenic activity is inappropriately augmented in E2-treated males.

The gluconeogenic findings contrast the typical role of E2 in increasing lipid beta-oxidation in females. Liver gene expression of carnitine palmitoyl transferase 1a (Cpt1a), which encodes for carnitine palmitoyl transferase 1a, the rate-limiting step in mitochondrial fat oxidation, was increased with E2 in females (*p* = 0.031) but not in males (Fig. 3I). This is despite the presence of an estrogen response element (ERE) on the promoter of the Cpt1a, which would be expected to lead to increased expression in E2-treated males versus placebo males, as it does in females. To assess fuel utilization in vivo, we utilized indirect calorimetry. E2 treatment increased respiratory exchange ratio (RER) in E2-treated males versus females during both night and day cycles (*p* = 0.0066, *p* = 0.0023) (Fig. 3J). E2-treated males trended toward increased RER than placebo males during both night and day cycles (*p* = 0.1034, *p* = 0.1070). This suggests that E2 in males alter fuel preference toward carbohydrates rather than fats as a fuel, and it could indicate that augmented lipid utilization seen with E2 treatment in females is not conserved in hypogonadal males. Molecular analysis via snRNA seq found that Cpt1a gene expression was down-regulated in E2-treated males compared to placebo males (Fig. 3K). Sequencing data also revealed that E2 treatment in biological males down-regulated gene expression of other genes important for beta-oxidation, including acyl CoA oxidase 1 (Acox1) and 2 (Acox2). Collectively, this data suggested that estrogenic pathways in males might impair liver fat utilization as an energy source, perhaps leading to augmented gluconeogenesis for carbohydrate fuel supply and utilization.

### E2-treatment in males modify subcutaneous adipose morphology toward hypertrophy and potential TNFα-driven inflammation

**Fig. 4:**
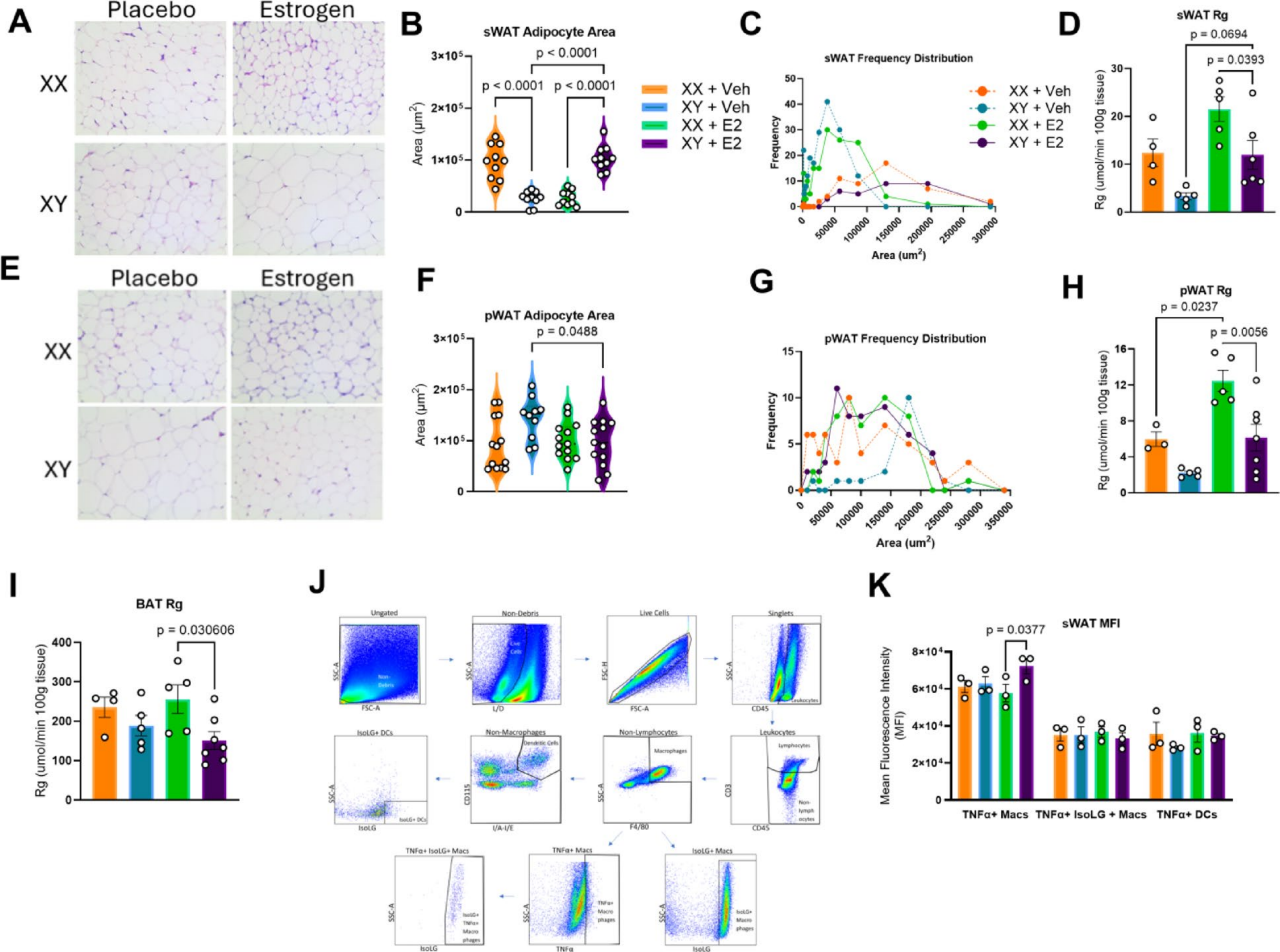
E2-treated hypogonadal males have increased sWAT area per adipocyte and reduced pWAT area but exhibit signs of sWAT hypertrophy. **A** Representative H&E subcutaneous adipose images. **B** sWAT adipocyte distribution of subcutaneous adipocyte size. n = 10, One-way ANOVA with Tukey’s Multiple Comparison Test. **C** sWAT frequency distribution of individual adipocyte areas. n = 10. **D** Euglycemic-hyperinsulinemic clamp of tissue-specific glucose uptake (Rg) in sWAT demonstrates increased glucose uptake in E2-treated males versus placebo males. n = 3 = 5, One-way ANOVA with Tukey’s Multiple Comparison Test. **E** Representative histological images of perigonadal adipose tissue via **H**, **E**. **F** pWAT adipocyte area shows increased area in placebo males versus E2-treated males. n = 10, One-way ANOVA with Tukey’s Multiple Comparison Test. **G** pWAT Frequency distribution of individual adipocyte areas. n = 10. H) Euglycemic-hyperinsulinemic clamp of tissue-specific glucose uptake (Rg) in pWAT shows that glucose uptake was increased in pWAT of E2-treated females versus placebo females and E2-treated females versus E2-treated males. n = 3 = 5, One-way ANOVA with Tukey’s Multiple Comparison Test. **I** Euglycemic-hyperinsulinemic clamp of tissue-specific glucose uptake (Rg) in brown adipose tissue (BAT) is increased by E2 in females. n = 3 = 5, One-way ANOVA with Tukey’s Multiple Comparison Test. **J** sWAT Flow Cytometric Gating Strategy. **K** IFN-γ mean fluorescence intensity is increased in placebo males versus E2-treated males. MFI is increased in macrophages of E2-treated males versus females. n = 3; Two-way ANOVA with Tukey’s Multiple Comparisons Test

Estrogens are known to promote deposition of fats into sWAT depots in biological females, with a metabolically favorable preference for adipocyte hyperplasia rather than hypertrophy. We sought to determine the influence of long-term E2 administration on the morphology of sWAT and visceral perigonadal white adipose tissue (pWAT) in hypogonadal males. Histology of sWAT and subsequent analysis revealed an increase in individual adipocyte area in E2-treated males compared with placebo males (*p* < 0.0001) (Fig. 4A,B). This trend was reversed in biological females, where placebo females’ adipocyte area was larger than that of E2-treated females (*p* < 0.0001). Frequency distribution demonstrated a trend toward increased adipose area and reduced total adipocyte number in the E2-treated males compared to the placebo males (Fig. 4C).During the hyperinsulinemic-euglycemic clamp, glucose uptake by subcutaneous fat was significantly higher in E2-treated females than in E2-treated males (*p* = 0.0393), with a trend toward increased glucose uptake with E2 treatment in males (*p* = 0.0894) (Fig. 4D).

Histology of visceral adipose revealed increased adipocyte area in placebo males versus E2-treated males (Fig. 4E). Individual adipocyte area of perigonadal adipose was significantly decreased in E2-treated males versus placebo males, with no differences between biological female groups (*p* = 0.048) (Fig. 4F). There was an increased distribution of large visceral adipocytes within placebo males compared to E2-treated males (Fig. 4H). Glucose uptake by perigonadal fat during the hyperinsulinemic-euglycemic clamp was significantly increased in with E2-treatment in females but not in males (*p* = 0.0237) (Fig. 4J).

Glucose uptake by brown adipose tissue was quantified during the hyperinsulinemic-euglycemic clamp to provide further insight into preferential depots for storage of excess energy. Brown adipose tissue uptake of glucose was significantly higher in E2-treated females than in E2-treated males (*p* = 0.0306) (Fig. 4L).

To establish if subcutaneous adipocyte hypertrophy in E2-treated males resulted in a pro-inflammatory phenotype, flow cytometric analysis of immune cells and cytokines within sWAT was conducted. E2-treated males exhibited increased TNFα content within adipose-resident macrophages when compared to E2-treated females (*p* = 0.0348) (Fig. 4I).

### Improved HDL uptake with E2 treatment but blunted lipase activity and VLDL clearance

**Fig. 5:**
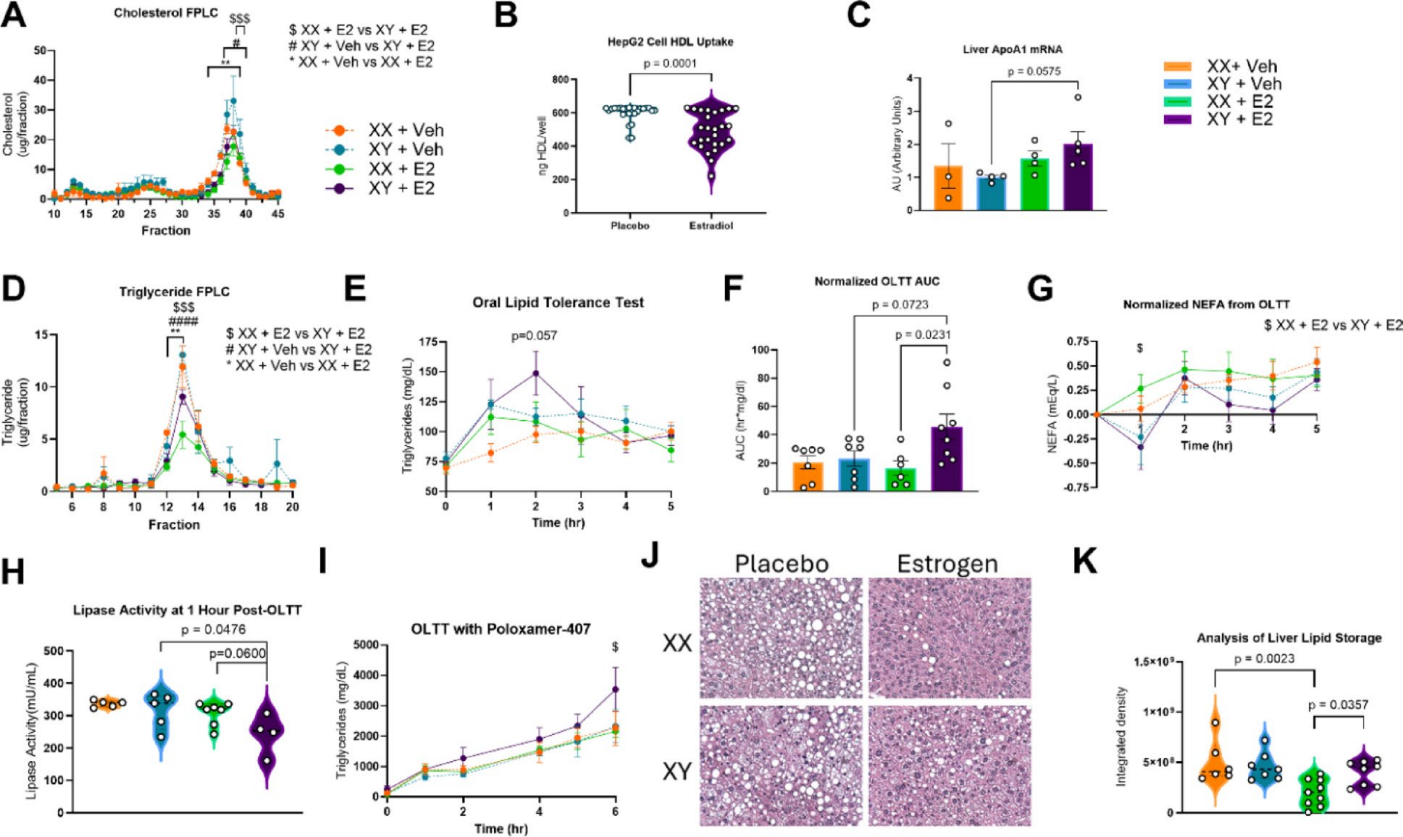
Favorable Cholesterol Changes but Unfavorable Modifications to Triglyceride Pathways in Estrogenized Males. **A** FPLC and cholesterol analysis of serum from huCETP transgenic mice. n = 3; Two-way ANOVA with Tukey’s Multiple Comparisons Test. **B** HepG2 cells treated for one week with 10 nM E2 exhibited significantly increased HDL uptake than vehicle-treated cells. n = 25, Mann–Whitney U-Test. **C** qPCR quantification of mRNA liver expression of Apoa1. n = 3–5; One-way ANOVA with Bonferroni’s Multiple Comparison Test. **D** FPLC and triglyceride analysis of serum from huCETP transgenic mice. n = 3; Two-way ANOVA with Tukey’s Multiple Comparisons Test. **E** Oral lipid tolerance test (OLTT) via olive oil gavage in 5 h fasted mice, with TGs taken via tail vein collection for 5 h. n = 5–9; Two-way ANOVA with Tukey’s Multiple Comparisons Test. **F** Area under the curve analysis from the OLTT using normalization of each sample to baseline. n = 5–9, One-way ANOVA with Bonferroni’s Multiple Comparison Test. **G** NEFA levels during the OLTT using tail vein blood collection. n = 5–9, One-way ANOVA with Bonferroni’s Multiple Comparison Test. **H** Lipase activity from OLTT at 1-h post gavage shows significantly reduced lipase activity in E2-treated males versus placebo males. n = 4–7, One-way ANOVA with Bonferroni’s Multiple Comparison Test. **I** Poloxamer 407 administered via IP injection, followed immediately by OLTT in five hour fasted mice. At 6 h post OLTT, E2-treated males have significantly increased TG levels when compared to E2-treated females. n = 4; Two-way ANOVA with Tukey’s Multiple Comparisons Test. **J** Histological representation of liver lipid droplet accumulation from H&E. **K** ImageJ analysis of liver lipid droplet density reveals increased lipid droplet density in E2-treated males than E2-treated females. n = 6–9; One-way ANOVA with Bonferroni’s Multiple Comparison Test

Lipoprotein profile changes alter CVD risk, with increased HDL concentrations contributing to reduced risk and higher levels of circulating VLDL contributing to increased risk. We analyzed cholesterol concentration within the serum of transgenic mice containing humanized cholesterol ester transfer protein (huCETP), as these mice possess higher levels of LDL and VLDL than wild-type mice, resulting in a more human-like lipoprotein profile. E2-treated, androgen-deprived males exhibited a lower cholesterol peak in their HDL subfraction than did the placebo males (Fig. 5A). Likewise, placebo females had a higher cholesterol peak in the HDL subfraction than did E2-treated females.

To extend these findings to human tissue, HepG2 cells derived from a young adult male were exposed to 10 nM E2 or placebo for one week before HDL uptake was quantified. E2-treated HepG2 cells had significantly increased HDL uptake than did placebo-exposed cells, further supporting that E2 treatment benefits HDL composition and function in males (Fig. 5B). To probe HDL lipoprotein production, we returned to the animal model and found that ApoA1 mRNA expression in the liver trended toward being increased with E2 treatment in androgen-deprived male mice (Fig. 5C).

To quantify changes to triglyceride concentration in VLDL, FPLC and triglyceride analyses were performed on huCETP mice. It showed that E2-treated males had higher peak triglycerides in their VLDL subfraction than E2-treated females (Fig. 5D). An oral lipid tolerance test (OLTT) was conducted to provide insight into triglyceride (TG) handling. E2-treated males had a higher peak TG level than E2-treated females (Fig. 5E). Overall area under the curve showed significantly higher lipid excursion in the E2-treated males than females, despite both sexes receiving the same amount of oil via oral gavage (Fig. 5F). There was a trend toward higher excursion in E2-treated males compared to placebo males. Possible causes of increased TG excursion in the OLTT included decreased lipase activity with reduced non-esterified fatty acid (NEFA) levels and/or reduced non-lipase lipoprotein receptor clearance. NEFA levels were analyzed following the oil challenge and were found to be significantly higher at 1-h post-gavage in E2-treated females than in E2-treated males (Fig. 5G). Lipase activity at this time point showed significantly lower lipase activity in E2-treated males than in placebo males (*p* = 0.0476), with a trend toward decreased activity in E2-treated males compared to E2-treated females (*p* = 0.060)(Fig. 5H). The surfactant poloxamer-407 was administered prior to the OLTT to determine how efficiently VLDL could be produced by the liver and cleared by non-lipase receptor activity. E2-treated males had significantly higher levels of TGs at 6 h post-OLTT than did E2-treated females, with a trend toward higher levels in E2-treated males than placebo males (*p* = 0.0904) (Fig. 5I).

Altered liver secretion of lipoproteins, as well as blunted beta-oxidation, can impact liver lipid accumulation and the development of metabolic-associated fatty liver disease. We quantified lipid droplet density in liver histological samples (Fig. 5J). E2-treated females had lower lipid droplet density than placebo females (*p* = 0.0023) (Fig. 5K). E2-treated males had higher liver lipid density than E2-treated females (*p* = 0.0357), and lipid density was not significantly different in E2-treated males than in placebo males.

### E2 Favorably modifies liver genes in HDL pathways in males and females but adversely modifies VLDL production and clearance pathways in males

**Fig. 6:**
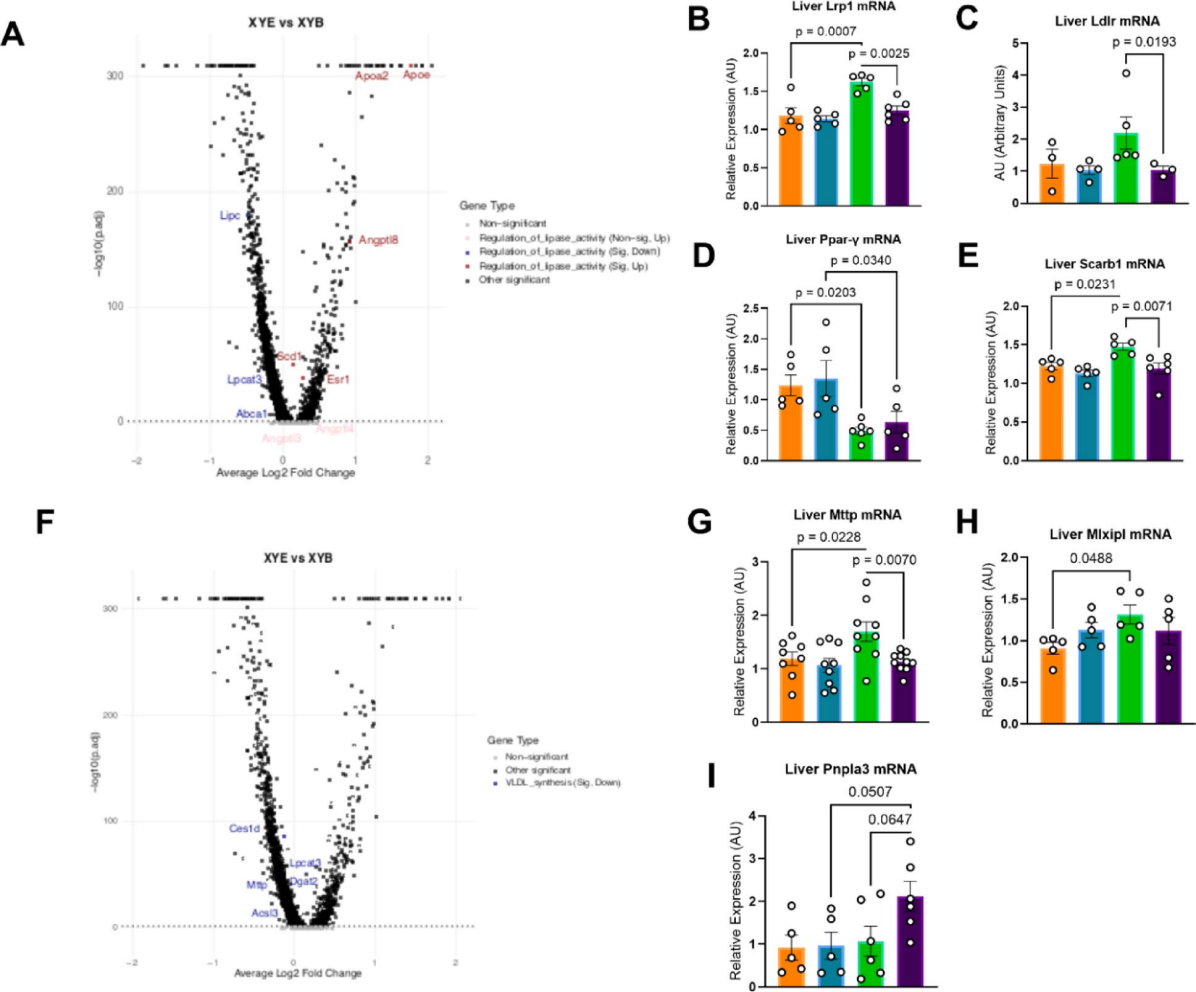
E2 Treatment Adversely Alters Liver Lipase and VLDL Pathways in Males. **A** Volcano plot representation of liver snRNA sequencing analysis of lipase activity pathways altered by E2 treatment in biological males. Significantly up regulated: *Esr1, Apoa2, Angplt8, Scd1, Apoe*. Significantly downregulated: *Lipc, Lpcat3, Abca1*. n = 3; Multiple Testing with Benjamini Hochberg False Discovery Rate (BH/FDR). **B** Lrp1 liver mRNA expression is reduced in E2-treated males versus E2-treated females and increased with E2-treatment in females. n = 4–5; One-way ANOVA with Bonferroni’s Multiple Comparison Test. **C** Liver qPCR quantification of Ldlr reveals a trend toward reduced mRNA expression in E2-treated males versus E2-treated females. n = 4–5; Kruskal–Wallis Test with Dunn’s Test for Multiple Comparisons. **D** Ppar-γ liver mRNA expression is reduced with E2-treatment in both males and females. n = 4–5; One-way ANOVA with Bonferroni’s Multiple Comparison Test. **E** Liver qPCR quantification of Scarb1 shows increased expression with E2 treatment in females but not in males, with significantly increased expression in E2-treated females than E2-treated males. n = 4–5; One-way ANOVA with Bonferroni’s Multiple Comparison Test. **F** Volcano plot representation of liver snRNA sequencing analysis of VLDL pathways altered by E2 treatment in biological males. Significantly down-regulated:*Ces1d, Mttp, Lpcat3, Dgat2, Acsl3*. n = 3, Multiple Testing with BH/FDR. **G** Mttp liver mRNA expression is increased with E2-treament in females and is higher in E2-treated females than in E2-treated males. n = 5; One-way ANOVA with Bonferroni’s Multiple Comparison Test. n = 8–9, One-way ANOVA with Bonferroni’s Multiple Comparison Test. **H** Mlxipl liver mRNA expression in increased with E2 treatment in females but not in males. n = 5; One-way ANOVA with Bonferroni’s Multiple Comparison Test. **I** Liver mRNA expression of Pnpla3 trends higher with E2 treatment in males and in E2-treated males versus E2-treated females. n = 5; One-way ANOVA with Bonferroni’s Multiple Comparison Test. All data in this figure was generated using C57Bl/6 mice

Molecular changes in hepatic lipid pathways were examined by qPCR and sRNA seq. Lipase‑pathway analysis showed that E2 treatment reduced hepatic expression of Lipc (the gene encoding hepatic lipase (HL)) (Fig. 6A) and increased the expression of several lipase inhibitors, notably Angptl8, a potent LPL inhibitor, and ApoA2, an HL inhibitor. There were also trends toward increased expression of Angptl3 and Angptl4 levels. These alterations altogether could underlie the reduced maximal lipase activity observed in E2‑treated males during the OLTT.

Optimal Apo‑E binding to LRP1 and LDLR requires lipidation by ABCA1, whose hepatic expression was also diminished by E2 in males. qPCR revealed a trend toward estrogen‑induced up‑regulation of Lrp1 in females but not in males (*p* = 0.0007) (Fig. 6B), and Ldlr expression was increased by E2 treatment only in females, resulting in significantly higher mRNA levels in E2‑treated females versus males (*p* = 0.0193; Fig. 6C). Liver peroxisome proliferator gamma (PPAR-γ) mRNA expression was reduced with E2 treatment in both males and females, which is an expected effect of E2 treatment, but decreased expression could reduce PPAR-y from positively regulating VLDL clearance mechanisms in E2-treated males (*p* = 0.0340) (Fig. 6D). Additionally, Scarb1 expression was lower in E2‑treated males (*p* = 0.0071) (Fig. 6E). This suggests that while physiological findings point toward VLDL clearance pathway impairment in E2-treated males, there could also be reduced activation of HDL‑related genes that are estrogen‑responsive in biological females.

E2 treatment in hypogonadal males down-regulated gene expression of hepatic genes in the VLDL production pathway, including Dgat3, Lpcat3, Ces1d, and Acsl3, further strengthening the molecular hypothesis of VLDL pathway alterations in E2-treated males (Fig. 6F). qPCR showed that mRNA expression of Mttp was increased with E2 in females but not in males (p = 0.0228) (Fig. 6G). snRNA seq data similarly showed reduced mRNA levels of Mttp in estrogen-treated biological males compared with placebo males (Fig. 6F). As Mttp encodes for microsomal triglyceride transfer protein (MTP), the rate-limiting step in VLDL production, this data could point toward MTP involvement in VLDL pathway impairment. E2-treated females, dissimilarly from E2-treated males, exhibited increased expression of Mxlipl, a lipogenic transcription factor, compared to placebo females (p = 0.0488) (Fig. 5H). Additionally, E2-treated males had increased liver mRNA expression of Pnpla3 when compared to placebo males (Fig. 6I). Increased Pnpla3 could reduce lipase access to breakdown lipid droplets within hepatocytes and further contribute to the observed phenotype.

## Materials and methods

All animal work complied with the NIH Guide for the Care and Use of Laboratory Animals and was approved by the Vanderbilt University Institutional Animal Care and Use Committee (IACUC) [28].

### Animals and experimental design

Eight‑week‑old male C57BL/6 J mice and huCETP‑transgenic (B6.CBA Tg(CETP) 5203Tall/J) mice on a C57BL6/J background (Jackson Laboratory, Stock #003904) were used. Mice were housed at 22 ± 1 °C on a 12‑hour light/12‑hour dark cycle with ad‑libitum chow until two weeks after gonadectomy (see supplemental Fig. 1A). Baseline (pre‑E2) data were collected at nine weeks of age. Thereafter, mice were switched to a western‑style diet (Research Diets #D12079B) and kept at thermoneutrality (29 ± 1 °C, 40 ± 10% humidity, 12‑hour light/12‑hour dark) for nine weeks beginning two weeks post‑gonadectomy to induce metabolic dysfunction [29]. Mice were euthanized at 21 weeks via CO_2_ inhalation and exsanguination. Tissues were flash-frozen in liquid nitrogen and then stored at –80 °C for molecular analysis or were fixed in 10% neutral‑buffered formalin for histology. Unless otherwise noted, blood was obtained by cardiac puncture, collected into 10% EDTA‑containing tubes, immediately placed on ice, centrifuged at 2,000 × g for 10 min at 4 °C, and the plasma supernatant was aliquoted and stored at –80 °C for subsequent assays.

For the snRNA seq experiments, a separate cohort schematic was utilized. C57BL/6 mice were gonadectomized at 11 weeks. They were placed on high fat diet (Research Diets #D12492) at 33 weeks, when estradiol benzoate dissolved in sunflower oil, or sunflower oil for vehicle-treated mice, was administered intraperitoneally every four days from week 33 to week 47. Mice were sacrificed at 47 weeks and tissues were harvested for snRNA sequencing analysis.

### Gonadectomy (Gdx)

All surgeries were performed under inhaled isoflurane (1–5% in 100% O₂, 1–2 L min⁻^1^) [30]. The surgical field was disinfected with 0.2% chlorhexidine prepared from a 1:1 solution of 0.4% chlorhexidine gluconate (Hibiclens, #57,532) and deionized water.

*Male orchiectomy* – A midline scrotal incision was made. The tunica vaginalis was opened, the vas deferens was cauterized, and both testes were excised. For sham operation, the scrotal incision was made, the tunica was exposed, and then the incision was sutured close. This procedure follows the protocol described by Smith et al. (2001) [23].

*Female ovariectomy* – A dorsal midline skin incision was made, the peritoneum was opened, and both ovaries were exteriorized, ligated, and removed. The technique is follows the protocol reported by Zhu et al. (2013) [15].

After gonadectomy, a sub‑cutaneous pocket was created over the flank using blunt dissection to separate fascia and either a β‑estradiol pellet (Innovative Research, NE‑121, 0.25 mg, 90‑day release) or a vehicle pellet (Innovative Research, NC‑111) was implanted. Post‑operative analgesia consisted of ketoprofen (5–10 mg kg⁻^1^) administered pre‑operatively and every 24 h for three days, plus a single prophylactic dose of ceftriaxone (15–25 mg kg⁻^1^) the day of the surgery. Estradiol levels were confirmed at sac with Thermofisher Rapid ELISA assay (EELR013) using blood collected via cardiac puncture in tubes containing 10% EDTA, from which plasma was collected following centrifugation (Supplemental Fig. 3C).

### Treatment groups

Four groups were generated (see Supplemental Fig. 1B). XY + E2: Gonadectomized males receiving an estradiol pellet. XY + Veh: Gonadectomized males receiving a vehicle pellet. XX + E2: Gonadectomized females receiving an estradiol pellet. XX + Veh: Gonadectomized females receiving a vehicle pellet. A fifth group, XY + Sham (Sham-operated males receiving a placebo pellet), was also generated for a preliminary set of glucose metabolism studies included in the supplement (Supp. Figure 7).

### Body‑composition measurement

Whole‑body fat and lean mass were measured at 07:00 h using a Bruker Minispec mq Series time‑domain nuclear magnetic resonance (NMR) analyzer (frequency range 5–60 MHz).

### Metabolic tolerance tests

All tolerance tests were performed after a five‑hour fast (07:00–12:00 h).

*Oral Glucose Tolerance Test (OGTT).* Glucose (2 g kg⁻^1^ lean mass, 20% glucose solution) was administered by oral gavage. Blood glucose was measured with a Contour Next EZ glucometer at –10, 0, 5, 10, 15, 20, 30, 45, 60, 90, and 120 min [23]. Plasma insulin at baseline and 15 min was quantified by ELISA (CrystalChem #90,080). Advanced glycation end‑product (AGE) levels were measured using an ELISA kit (Abcam #238,539) at baseline and 2 h post‑gavage.

*Oral Lipid Tolerance Test (OLTT)*. As described by Ayala, et al. (2010) [31], following a 5 h fast, mice received 200 µL olive oil (Good Value, UPC#078742075198) by oral gavage. Tail‑vein blood was collected at –10, 0, 60, 120, 180, and 240 min. Triglycerides were measured with Infinity™ Triglyceride Liquid Stable Reagent (ThermoFisher #TR22421). One hour after the OLTT, serum lipase activity was assessed (Sigma Aldrich #MAK048). For the Poloxamer‑407‑enhanced OLTT, mice were injected intraperitoneally with 200 µL of 25 mg mL⁻^1^ Poloxamer‑407 (Millipore‑Sigma #16,758) prior to oil gavage; triglycerides were then quantified with the Novus Biologicals kit (NBP3‑24,540). Lipid hydroperoxides were measured using ELISA kit for malondialdehyde (Sigma Aldrich #MAK568) on tissues harvested at 6 h post-OLTT and poloxamer challenge.

*Intraperitoneal Insulin Tolerance Test (ITT)*. Insulin (Eli Lilly, Humulin R, Stock # R-100) was injected intraperitoneally at 1 U kg⁻^1^ lean mass; glucose was measured at 15, 30, 60, and 90 min, as described by Smith et al. (2001) [23]. If glucose fell below 45 mg dL^−1^ at any point, 10% dextrose was administered via oral gavage, and the mouse was excluded from the study.

*Intraperitoneal Pyruvate Tolerance Test (PTT)*. Sodium pyruvate (Sigma #P2256) was injected intraperitoneally at 2 g kg^−1^after a 6‑hour fast; glucose was measured at baseline and 15, 30, 45, 60, and 120 min, as described by Smith et al. (2001) [23].

### Cell‑culture experiments

Human HepG2 cells (ATCC, HB‑8065) were maintained in RPMI‑1640 supplemented with 10% charcoal‑stripped fetal bovine serum (Sigma F6765). Cells were treated with 10 nM water‑soluble 17‑β‑estradiol (Sigma #E4389) for seven days, as described by Zhu, et al. (2013) [7]. HDL uptake was quantified using an HDL Uptake Assay (Abcam, #ab204717) according to the manufacturer’s protocol.

### Fast‑performance liquid chromatography (FPLC)

Plasma lipoproteins were separated on a Superose 6 column (GE Healthcare) using an FPLC system. Triglyceride and cholesterol concentrations in each fraction were measured with Infinity™ Triglyceride (ThermoFisher #TR22421) and Infinity™ Cholesterol (ThermoFisher #TR13421) reagents, respectively, using the protocol described in DeSmet et al. (2017) [32].

### Single‑nucleus RNA‑sequencing (snRNA‑seq)

Liver tissue from was harvested and isolated using the Invent Nuclei Isolation Kit (Cat #NI‑024) and processed with the 10 × Genomics Chromium Single‑Cell 3′ V3.1 workflow. Libraries were sequenced on an Illumina NovaSeq S4 (2 × 100 bp) to ~ 20 k reads per nucleus.

Raw FASTQ files were aligned with Cell Ranger v6.0.0 [33], ambient RNA was removed using CellBender [34], and downstream analysis was performed in Seurat v4 employing the dictionary‑learning integration method [35]. Cell‑type annotation relied on canonical liver markers from the mouse cell atlas [36] and spatial proteogenomics liver‑macrophage reference [37]. Differential expression between estradiol‑treated and control nuclei was assessed with Seurat’s Wilcoxon rank‑sum test (Bonferroni‑adjusted *p* < 0.05) [38].

### Flow cytometry

Aortas were dissected, minced, and digested in 1 mg mL⁻^1^ Collagenase A (Sigma #10,103,578,001) plus Collagenase B (Sigma #11,088,807,001) and DNase I (ThermoFisher #EN0521) using the protocol described by Kirabo et al. (2014) [39]. Subcutaneous (sWAT) and perigonadal visceral white adipose tissue (pWAT) depots were similarly digested with 1 mg mL⁻^1^ Collagenase Type 1 (LS Biosciences #LS004196). After filtration and red‑blood‑cell lysis, cells were stained with the following antibodies (all from BD Biosciences unless noted):

Macrophage panel: CD45 (Pacific Orange), CD3 (BV‑606), MerTK (BV‑480), F4/80 (BV‑785).

Dendritic‑cell panel: CD45 (Pacific Orange), CD3 (BV‑606), MerTK (BV‑480), I‑A/I‑E (SF‑532), CD115 (PE‑efluor 610).

Intracellular cytokines (IL‑6, TNF‑α) were detected after fixation/permeabilization (Invitrogen kit) using PerCP‑anti‑IL‑6 and BV‑711‑anti‑TNF‑α. Data were acquired on a BD FACSCanto II and analyzed with FlowJo (live‑cell singlet gating, fluorescence‑minus‑one controls).

#### Portal‑vein insulin injection (± insulin‑tissue collection)

Mice fasted for 5 h (07:00–12:00) were anesthetized with isoflurane, a ventral midline skin flap was created, and the portal vein was exposed. Insulin (0.75 U kg⁻^1^ lean mass) was injected directly into the portal vein using a 28‑gauge needle held in place with a bulldog clamp for three minutes. The right medial liver lobe was excised, flash‑frozen, and the remaining lobes were collected. In a parallel cohort, insulin (2.5 U kg⁻^1^) was administered intraperitoneally 15 min before sacrifice.

#### Hyperinsulinemic‑euglycemic clamp

Five days after catheter implantation, clamps were performed in five‑hour‑fasted mice. A primed (5.4 µCi) continuous (0.135 µCi min⁻^1^) infusion of [^3^H]‑glucose began at *t* = ‑90 min (basal period), using the protocol described in Ayala et al. (2010) [31]. Hyperinsulinemia (4.0 mU kg⁻^1^ min⁻^1^ Eli Lilly, Humulin R) started at *t* = 0; blood glucose was measured every ten minutes and a 50% dextrose infusion was adjusted to maintain ~ 150 mg dL⁻^1^. At *t* = 120 min a bolus of [^14^C]‑2‑deoxyglucose was administered to assess tissue glucose uptake. Mice were euthanized at *t* = 155 min and tissues were flash‑frozen for analysis.

#### Coagulation assays

Plasma for coagulation factor assays was collected in 3% sodium citrate tubes at sacrifice via inferior vena cava stick. Circulating coagulation factors were assessed via ELISA: coagulation factor 7 (NOVUS Biologicals #NBP2-67,963), coagulation factor 8 (NOVUS Biologicals #NBP2-76,578), coagulation factor 10 (NOVUS Biologicals #NBP2-82,404), and thrombin (Abcam #ab230933).

#### Histological image analysis

*Adipocyte morphometry* – H&E‑stained sections were analyzed with the Adipocyte Tools plug‑in [40] for ImageJ (size filter 50–20 000 µm^2^; 10 dilations). Four non‑overlapping fields per mouse were quantified; mean cell area and number were calculated.

*ERα immunohistochemistry* – Liver sections were stained with anti‑ERα (Abcam #ab16660) and visualized with DAB. A blinded observer scored ERα‑positive hepatocytes and total hepatocyte count.

*Lipid‑droplet quantification* – Fiji/ImageJ macro: 8‑bit conversion → threshold (179‑255) → mask → watershed → particle analysis (size ≥ 900 px, circularity 0.45–1.00).

#### Statistical analysis

Data are presented as mean ± SEM. Normality was assessed with the Shapiro‑Wilk test. For normally distributed data, one‑way ANOVA with Bonferroni post‑hoc or two‑way ANOVA with Tukey’s multiple‑comparison test was used. Non‑parametric data were analyzed with Kruskal–Wallis Test with Dunn’s Test for Multiple Comparisons; pairwise comparisons employed an unpaired t‑test when appropriate. A Monte‑Carlo Permutation Test with 10,000 random draws was performed to generate the null distribution for the UpSet‑plot overlaps; the empirical p‑value was calculated as the proportion of random overlaps that were equal to or greater than the observed overlap. All analyses were performed using GraphPad Prism 10 (GraphPad Software, San Diego, CA). A two‑tailed *p* < 0.05 was considered statistically significant.

## Discussion

Our data reveal a coherent metabolic signature that distinguishes hypogonadal males receiving E2 from both placebo‑treated males and E2‑treated females. We observed: a pro‑inflammatory milieu and hypertrophy of sub‑cutaneous adipose tissue, dysregulated hepatic VLDL production and clearance, impaired peripheral triglyceride clearance after an oral lipid challenge, and insulin resistance coupled with a paradoxical increase in gluconeogenesis despite improved oral glucose tolerance. Collectively, the convergence of these pathways reveals a dysregulated metabolic environment that could elucidate how E2 treatment imparts CVD risk in hypogonadal males.

Chronic E2 treatment in androgen‑deprived biological men results in a preferential distribution of fat into subcutaneous depots, similar to E2’s known effects in women [41]. While a shift toward peripheral adipose distribution is typically favorable, the morphological changes that occur in the subcutaneous adipose of E2‑treated hypogonadal males in response to increased lipid‑storage demand are poorly understood [42]. Histologic analysis showed a significant increase in adipocyte area in E2‑treated males compared with placebo males and E2‑treated females. This points toward hypertrophic expansion rather than hyperplastic recruitment. Hypertrophic adipose remodeling is associated with a pro‑inflammatory phenotype, wherein enlarged adipocytes secrete chemokines that attract pro‑inflammatory macrophages, amplifying local cytokine production, dyslipidemia, and systemic insulin resistance [42, 43]. Flow‑cytometric analysis of subcutaneous white adipose tissue (sWAT) revealed that chronic E2 treatment in hypogonadal males—but not in E2‑treated females—markedly enriched intracellular TNF‑α within CD45⁺CD3⁻MerTK⁺F4/80⁺ macrophages. This occurs despite E2 similarly benefiting body composition in both sexes and suggests that seemingly favorable changes to lipid distribution may be counteracted by a hypertrophic response in males.

Visceral adipocytes from the perigonadal fat pad, however, were significantly smaller in E2‑treated males than in placebo males, likely reflecting a shift of lipid storage away from visceral depots. The immune consequences of reduced visceral adipocyte size in this model are not well understood; future work should explore whether alterations in adipocyte progenitor pools drive the subcutaneous hypertrophy observed in E2‑treated hypogonadal males. Additional studies could further define the contribution of adipose tissue to systemic dyslipidemia and CVD risk through adipokine profiling, thermogenic pathway analysis, and lipolysis assays.

Divergently regulated genes, in addition to uniquely enriched genes, likely account for the discordant effects of E2 in hypogonadal males versus females. Hepatocyte snRNA‑seq showed that E2 regulates the expression of 1,780 genes similarly in both females and hypogonadal males—far more than the number of genes that show opposite enrichment directionality between the sexes (Supplemental Fig. 6). This supports that many of E2’s hepatic effects are sex‑independent. In contrast, 362 genes exhibit divergent regulation in hypogonadal males versus females. Overall, 620 genes are uniquely altered by E2 in hypogonadal males, exceeding the number uniquely responsive in females. snRNA‑seq and qPCR demonstrated down‑regulation of key VLDL‑assembly genes (Ces1b, Dgat2, Acsl3, Mttp) in livers of E2‑treated males vs. females. Notably, *Mttp* expression was not increased in E2‑treated males despite the presence of an estrogen‑response element (ERE) in its promoter. This sex‑specific transcriptional response may reflect epigenetic silencing of ERE‑containing promoters in post‑pubertal males, a hypothesis supported by the parallel suppression of *Scarb1*, another divergently regulated ERE-bearing gene. Future studies could determine whether ERE regions in these metabolic genes are accessible to ERα binding in hepatocytes of hypogonadal males. Further, it is important to note that the single‑nucleus RNA‑seq dataset was obtained from an earlier cohort that used IP‑administered E2 and a slightly different high‑fat diet. Although the experimental parameters differ from the present study, the core hormonal manipulation, androgen deprivation + chronic estradiol, is comparable, allowing the transcriptomic findings to inform the mechanistic findings presented here. Future work will repeat single‑nucleus profiling in the current model to confirm the gene expression pathways under identical dietary and dosing conditions.

Human studies of androgen-deprived biological males show that long-term E2 treatment plus androgen deprivation induces hypertriglyceridemia up to 30% higher than androgen deprivation alone [14, 15, 44, 45]. In our study, we showed that E2 treatment down-regulated *Lipc* (hepatic lipase) gene expression in hepatocytes and increased *Angptl8* and *ApoA2*, both inhibitors of lipoprotein lipase (LPL). This molecular data coincides with physiologic findings: E2‑treated males displayed lower maximal LPL activity and a trend toward reduced TG clearance after an oral lipid tolerance test (OLTT). When Poloxamer‑407 was used to block lipase‑mediated TG hydrolysis, serum TGs remained markedly elevated in E2‑treated males, indicating impaired non‑lipase VLDL‑receptor clearance, which aligns with reduced hepatic *Lrp1* and *Ldlr* expression. This echoes a human study, which shows that the combination of estrogens in hypogonadal males reduced LPL activity 44]. These findings suggest that estrogen in hypogonadal males compromises both enzymatic and receptor‑mediated pathways for TG removal. Future work using a model with a humanized lipoprotein profile will be needed to determine how this dysregulation influences atherosclerotic lesion formation in E2‑treated hypogonadal males.

Studies of MtF individuals found that androgen deprivation and estrogen treatment resulted in insulin resistance via hyperinsulinemic-euglycemic clamp, despite no change to type 2 diabetes incidence [44, 46]. This picture of insulin resistance without hyperglycemia is similarly shown in our study of hypogonadal, E2-treated male mice. Although OGTT curves were modestly improved in E2‑treated males versus placebo, hyperinsulinemic‑euglycemic clamps revealed no enhancement of whole‑body insulin sensitivity and, strikingly, a significant increase in gluconeogenesis during the clamp in E2‑treated males versus placebo. Pyruvate tolerance testing confirmed heightened gluconeogenic capacity, accompanied by up‑regulation of *Pck1* (via mRNA and snRNA‑seq) and down‑regulation of gluconeogenesis repressors (*Lepr*, *Tcf7l2*, *Ptpn2*). Future studies are necessary to establish the mechanism through which PCK1 mRNA level and snRNA seq gene expression is increased in E2-treated hypogonadal males versus placebo males. Indirect calorimetry showed an elevated respiratory exchange ratio (RER) in E2‑treated males, indicative of a shift toward carbohydrate oxidation and reduced fatty‑acid β‑oxidation, which is supported by reduced *Cpt1a* expression. Consistent with this metabolic shift, E2‑treated males displayed a trend toward higher nocturnal energy intake (Supplemental Fig. 4), although the difference did not reach statistical significance. Together, this represents another divergent pathway: E2 treatment in females augments β‑oxidative pathways, but this effect is absent in hypogonadal males. These data point to possible estrogen‑driven dysregulation of hepatic gluconeogenesis that may exacerbate glycemic control in the setting of androgen deprivation. Future studies assessing mitochondrial fuel flexibility and function in both liver and heart are needed to further define the role of E2 in altering metabolic fuel utilization in hypogonadal males.

A pilot study compared sham‑operated male controls to E2-treated and placebo males for a set of glucose metabolism studies. In the oral glucose tolerance test (OGTT), sham‑operated XY males showed higher plasma glucose than placebo males at 0, 5, and 90 min (*p* < 0.05) and higher glucose than E2‑treated males at every time point (*p* < 0.01) (Supplemental Fig. 7A). Calculated HOMA‑IR values confirmed that sham and placebo males have comparable measures of insulin resistance, both of which are markedly higher than in E2‑treated males (*p* < 0.01) (Supplemental Fig. 7C). These data indicate that the surgical sham procedure results in comparable glucose metabolism to the placebo male. Ultimately, for the purposes of increasing statistical power, and to maintain focus on the hypogonadal male as the most relative control group, the sham male was utilized only for these pilot studies. Future work examining the interaction of testosterone, in addition to hypogonadism and E2 treatment, could assess how the sham male differs from E2-treated and placebo males with respect to cardio metabolism.

We hypothesized that long‑term estrogen enhances HDL‑mediated cholesterol efflux, but that chronic androgen‑deprivation may blunt this benefit and impair HDL function. In HepG2 cells (a human hepatoma line derived from a 15‑year‑old male), 10 nM E2 treatment increased HDL‑mediated cholesterol efflux. While this was a human hepatoma cancer line, it provides some insight into the effect of E2 on human hepatic function. We believe the resulting augmentation of uptake was potentially via activation of hepatic ERα, which is known to augment HDL function. Consistent with this, our laboratory previously showed that gonad‑intact male liver‑specific ERα knockout (LERKO) mice exhibit a markedly reduced estrogen‑stimulated increase in HDL uptake compared with wild‑type littermates, confirming the importance of hepatic ERα for reverse cholesterol transport in E2‑treated, gonad‑intact males. The present study extended those findings to a model of androgen deprivation. In hypogonadal males treated with E2, hepatic ApoA1 mRNA trended upward, and single‑nucleus RNA‑seq revealed a significant increase in ApoA1 expression relative to placebo. However, the same dataset showed a down‑regulation of Abca1, the transporter that loads cholesterol onto ApoA1. This mirrors a human study of gender‑affirming hormone therapy (GAHT) that reported reduced reverse cholesterol transport and lower ABCA1 expression in peripheral mononuclear cells [47]. Fast protein liquid chromatography (FPLC) of plasma demonstrated higher HDL‑subfraction cholesterol peaks in placebo males than in E2‑treated males; intriguingly, placebo males also displayed higher HDL peaks than placebo females. Because HDL‑C concentration does not necessarily reflect functional capacity, we examined additional markers. Hepatocyte Scarb1 (encoding scavenger receptor class B‑1) mRNA was lower in E2‑treated males than in E2‑treated females. Scarb1 contains an ERE but appears to be divergently regulated by E2 in males versus females. Taken together, these data suggest that while estrogen can stimulate certain HDL‑related genes, namely ApoA1, concurrent androgen deprivation may disrupt downstream steps, such as Scarb1, that are essential for functional HDL particles. Future work should assess HDL antioxidant capacity, cholesterol efflux from foam cells, and whole‑body reverse cholesterol transport in E2‑treated, hypogonadal males versus appropriate controls. Further studies in a primary human hepatocyte line could confirm and expand upon HepG2 findings.

E2 treatment caused a significant increase in left‑ventricular (LV) mass in hypogonadal WT male mice. This was also observed in huCETP mice (Supplemental Fig. 2C). Within the huCETP cohort, fractional shortening was reduced in E2-treated males versus placebo males (Supplemental Fig. 2A). This echoes the findings of a human cohort in which men receiving combined anti‑androgens + diethylstilbestrol (DES) showed greater positive LV circumferential strain (LV‑CS) [32]. Positive LV‑CS indicates reduced systolic shortening and is closely associated with LV hypertrophy and diastolic dysfunction. A study in male rats reported that androgen‑deprivation‑induced LV‑mass reduction was reversed by E2, which also produced concentric remodeling in hypogonadal E2-treated males [48]. The concordant directionality of the murine, ratite, and human data suggests that estrogen‑mediated signaling in the male heart promotes concentric remodeling, likely secondary to the metabolic disturbances we observed (elevated triglycerides, inflammation, and altered substrate availability). Importantly, LV mass following LV mass normalization to lean body mass, E2‑treated females also displayed an increase in LVM (Supplemental Fig. 3). This appears to reflect their markedly lower total body weight—E2‑treated females were the smallest group in the study—rather than true abnormal remodeling. This is supported by E2-treated male mice showing additional signs of cardiac dysfunction not evident in female mice, including increased lipid hydroperoxide accumulation in cardiac tissue following the surfactant and OLTT challenge as well as increased percent of aortic root CD45⁺CD3⁻MerTK⁺F4/80⁺ macrophages positive for lipid peroxidation byproduct isolevuglandin (IsoLG) (Supplemental Fig. 3B, D, E). These findings are preliminary but could indicate dysregulation of cardiac and aortic-resident macrophage lipid utilization in a process unique to E2-treatment in hypogonadal males More research is necessary to further investigate these changes and to establish a molecular mechanism through which these alterations occur.

E2‑treated males compared to placebo males exhibited larger LV end‑diastolic diameters (LVEDD) in the WT cohort and a trend toward reduced fractional shortening (p = 0.052) and ejection fraction (p = 0.1004) without LVEDD in the huCETP cohort (Supplemental Fig. 2A, E). Ejection fraction was significantly increased in E2-treated huCETP males compared to E2-treated females (p = 0.0221). Taken together, this supports a predominantly concentric phenotype, with a modest eccentric component observed only in wild-type mice. Although more studies are necessary to further examine cardiac function, the stronger directional congruence of cardiac abnormalities in the huCETP cohort with previous studies in humans and rats [32, 48] points toward the important role of VLDL and LDL in mediating increased cardiac harm in hypogonadal males given E2. Transgenic mice with the huCETP minigene have a more human-like lipoprotein profile, with decreased HDL and increased VLDL and LDL, rather than storing most circulating lipids in HDL, as mice naturally do [49]. As the investigation into cardiac function was preliminary, more research is necessary to establish a strong understanding of cardiac physiology in E2-treated males. To explore the cardiac phenotype further, future studies should consider the huCETP model with short‑axis and parasternal long‑axis echocardiographic analyses at early and late time points to clarify the balance between concentric and eccentric remodeling, potentially extend treatment time, provide strain analysis, and determine whether the observed LV enlargement translates into functional impairment.

For the treatment of metastatic PrCa, medical or surgical castration is indicated [50]. For this study, orchiectomy was selected to establish the biological effects of androgen depletion and estrogen supplementation without risk of off-target effects that could be seen with medical castration therapies. No difference in survival has been established between gonadotropin releasing hormone (GnRH) antagonists and surgical castration at 30 months [50]. Medical castration, including LHRH agonists and GnRH antagonists, are more commonly utilized in the treatment of metastatic PrCa than surgical castration [50]. Thus, the interaction between E2 treatment and medical castration therapies is an important area of future research to determine whether co-administration of GnRH antagonists amplify or mitigate the lipid, adipose, and glucose effects identified within this study.

For gender-affirming hormone therapy indications, mineralocorticoid receptor antagonist spironolactone or anti-androgen cyproterone acetate, for populations outside of the US, are the most common methods of androgen deprivation [22, 51]. To focus on this population, the combination of E2 with an MR antagonist or anti-androgen would best resemble the specific biological milieu found with GAHT.

Our study used a subcutaneous delayed-release E2 pellet for chronic estrogen exposure. Serum E2 was quantified by ELISA; however, ELISAs for E2 have limited sensitivity in the sub-physiological range typical of gonadectomized mice, making absolute values, especially in the placebo groups, difficult to interpret with confidence. Consequently, the significant differences we observed between E2-treated males and placebo males should be regarded as relative differences rather than precise quantitative estimates of circulating estradiol (Supplemental Fig. 3C). The significant increase in serum E2 levels in E2-treated versus placebo males, together with the lack of a significant difference between E2-treated males and females, confirms that the pellet successfully raised serum E2 levels in hypogonadal males. Importantly, the core physiological and mechanistic conclusions—impaired triglyceride clearance, heightened hepatic gluconeogenesis, adipose inflammation, and modest cardiac remodeling in E2-treated, androgen-deprived males—are supported by a comprehensive set of phenotypic readouts (clamp studies, tolerance tests, transcriptomics, and histology) that do not rely on exact serum E2 concentrations. Thus, despite the assay’s limitations, our findings remain robust and biologically meaningful. Future work employing mass spectrometry–based quantification could provide more precise quantification of serum E2 levels, particularly for the placebo groups.

Subcutaneous E2 administration via pellet was utilized rather than oral DES, to avoid the pronounced first‑pass hepatic exposure that drives increased hypercoagulability [4, 51]. While this route minimizes acute changes in coagulation factors, we did assess coagulation markers to ensure that potential increased hypercoagulability due to E2 was considered. There were no significant changes to coagulation factors VII, VIII, X, or thrombin between any of the groups (Supplemental Fig. 5). While outside the scope of this study, future investigation of platelet in-vivo thrombosis and coagulation factor activity would improve understanding of how coagulation dynamics are altered by estrogens and androgen deprivation.

In hypogonadal male mice, chronic E2 treatment produces a multifaceted metabolic disturbance characterized by unfavorable adipose morphology and inflammation, suppressed hepatic VLDL assembly coupled with defective TG clearance, enhanced gluconeogenesis despite normal insulin signaling, and selective improvements in HDL function. These alterations collectively provide a mechanistic basis for the heightened CVD mortality observed in androgen‑deprived men receiving estrogen therapy. Future work should delineate the epigenetic mechanisms underlying sex‑specific ERE silencing, further explore cardiovascular functional changes and fuel utilization, examine whether targeting VLDL‑remodeling pathways mitigates cardiac effects, and explore the interplay between estrogen and emerging androgen‑deprivation modalities. Such insights could enable the safe re‑introduction of estrogenic agents for prostate‑cancer treatment without incurring prohibitive cardiovascular risk.

## Conclusion

This comprehensive study showed that E2 treatment in hypogonadal males altered gluconeogenic regulation, increased subcutaneous adipocyte area and inflammatory profile, modified left ventricular structure, and reduced TG clearance through estrogenic modulation to VLDL pathways. These findings further our understanding of the physiologic and mechanistic changes to glucose and lipid metabolism that could mediate estrogen-induced CV harm in androgen-deprived males. We hope that future studies will build upon this work to clarify the molecular pathways responsible for observed left ventricular mass increases in E2-treated males, as well as target identified pathways and risk factors, such as adverse VLDL changes, adipose hypertrophy, and potential impaired metabolic flexibility. These discoveries could allow estrogens to once again serve as highly effective PrCa treatment without contributing to increased CVD mortality.

## Supplementary Information

Additional file1 (DOCX 2411 kb)

## Data Availability

The datasets used and/or analyzed during the current study are available from the corresponding author on reasonable request.
