# Supplementary material for: Sex‑specific cardiovascular risk in estrogen‑treated androgen‑deprived males: metabolic characterization of glucose, adipose, and lipid pathways
Source: Cardiovasc Diabetol. 2026 Feb 2;25:72. doi: 10.1186/s12933-025-03059-y (PMC12955195; doi:10.1186/s12933-025-03059-y)
Supplement: Supplementary file 1 — Additional file1 (DOCX 2411 kb) [file 12933_2025_3059_MOESM1_ESM.docx]

Supplemental Data


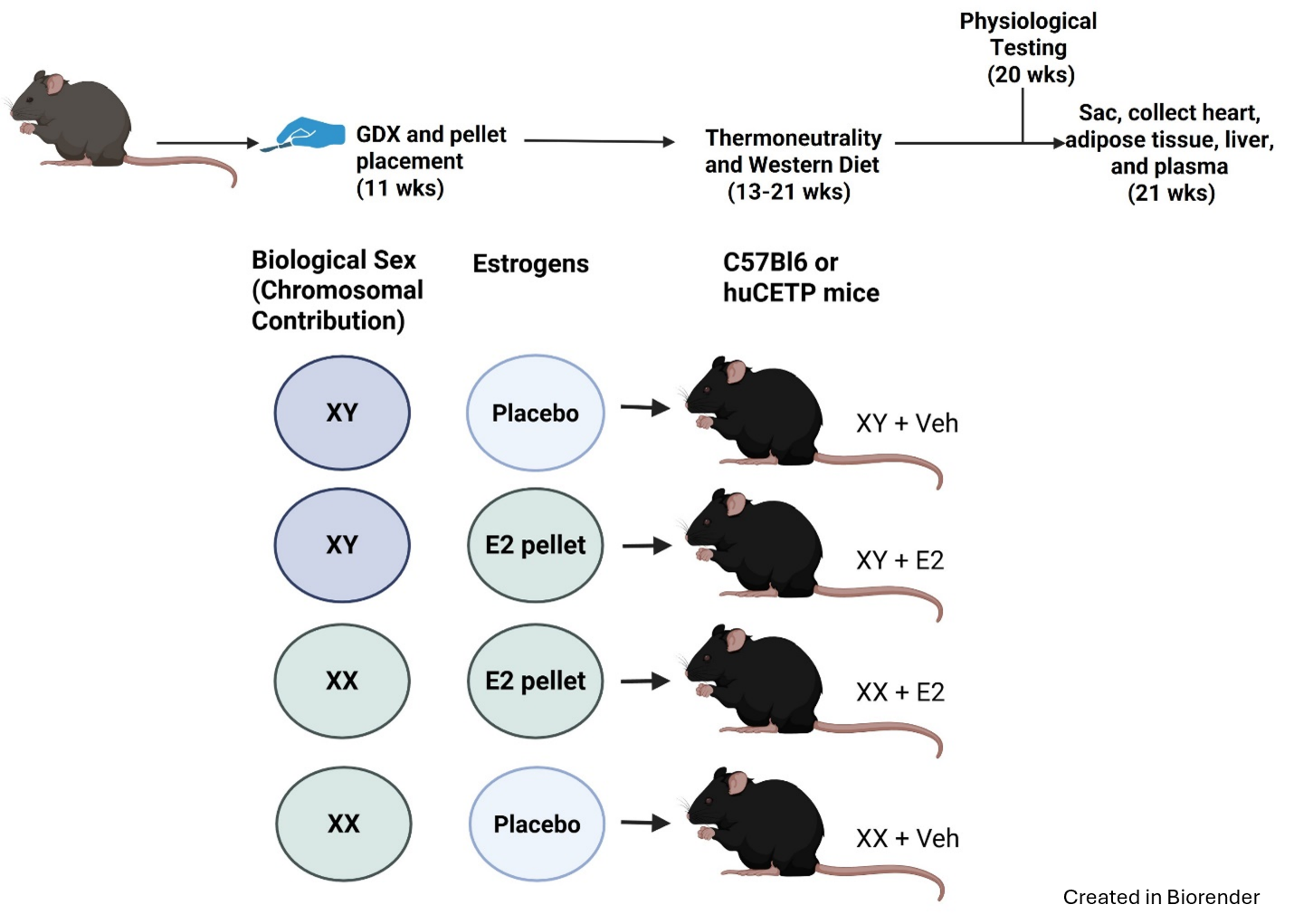


Supplemental Figure 1. Experimental Timeline and Experimental Mouse Model Diagram. A) Experimental timeline. B) Experimental diagram. Mice are C57Bl/6 unless otherwise noted.


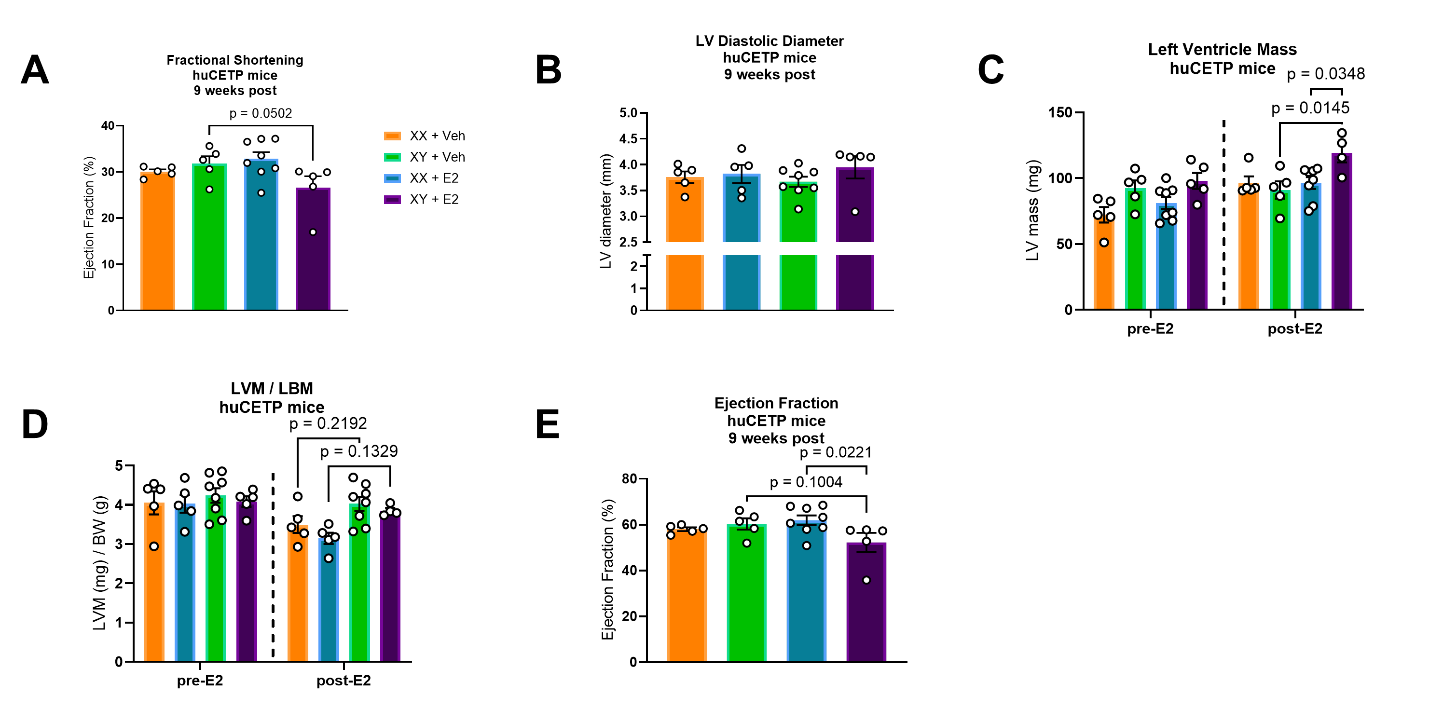


Supplemental Figure 2. huCETP Mouse Echocardiography Reveals Trend Toward Reduced Fractional Shortening (FS). A) Short-axis echocardiography at 20 weeks shows trend toward decrease in FS. n=5-8; Kruskal-Wallis Test with Dunn’s Test for Multiple Comparisons. B) Left ventricular end diastolic diameter was not different between huCETP groups at 20 weeks. n=5-8; Kruskal-Wallis Test with Dunn’s Test for Multiple Comparisons. C) Left ventricular mass at 20 weeks is increased in huCETP E2-treated male mice, relative to both placebo males and E2-treated females. n=5-8; Two-way ANOVA with Tukey’s Multiple Comparisons Test. D) LVM was normalized to lean body mass, and there was a trend toward increased LVM in E2-treated males versus placebo males. n=5-8; Two-way ANOVA with Tukey’s Multiple Comparisons Test. E) Ejection fraction at 20 weeks was significantly reduced in huCETP E2-treated males than in E2-treated females, with a downward trend relative to placebo males. n=5-8; Kruskal-Wallis Test with Dunn’s Test for Multiple Comparisons. All mice were huCETP Cre+.


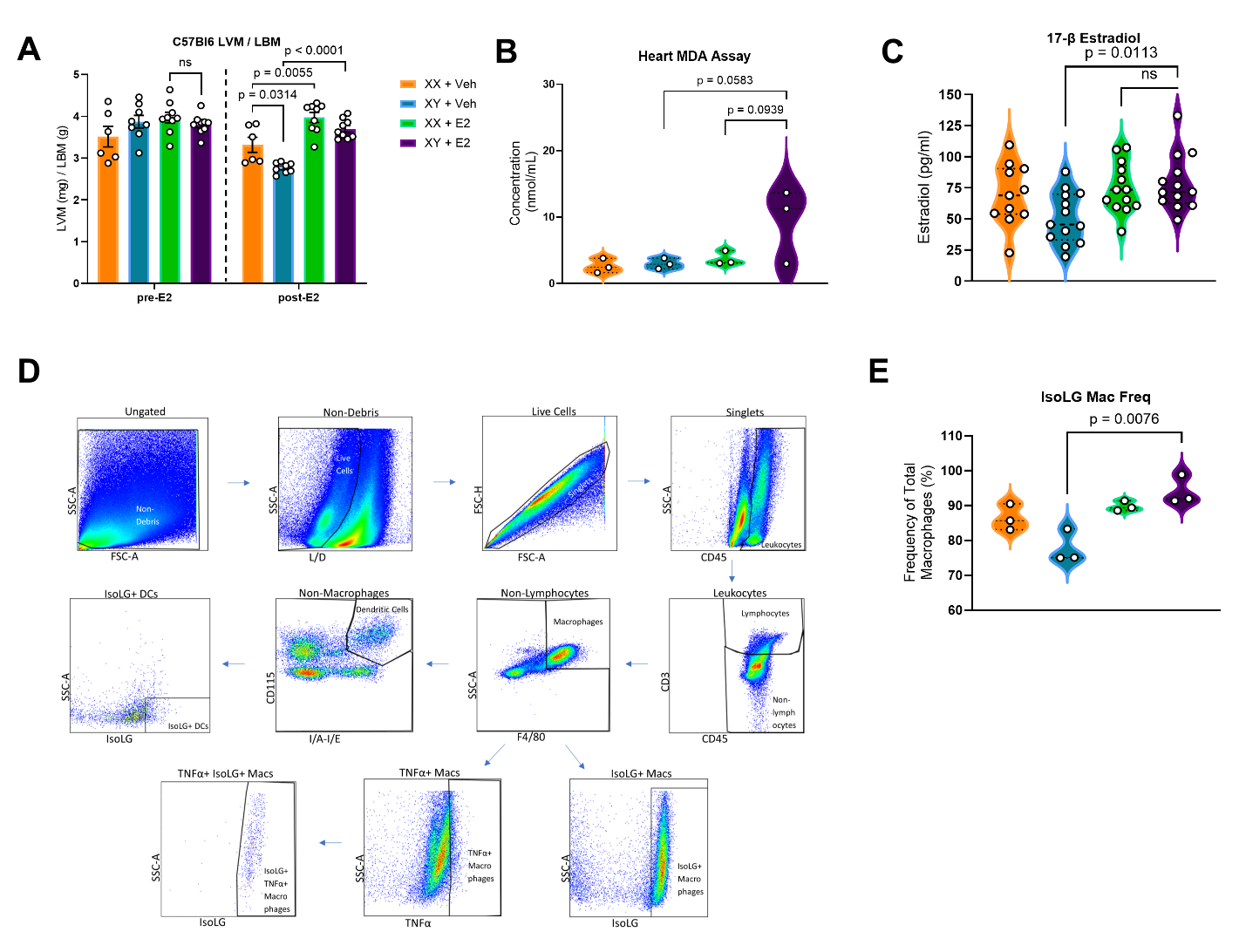


Supplemental Figure 3. E2 Levels and LVMi is Increased in C57Bl/6 E2-Treated Males with Cardiac and Aortic Macrophage Lipid Peroxidation. A) LVM was normalized to lean body mass, and normalized LVM was significantly increased in E2-treated males versus placebo males and E2-treated females versus placebo females. n=6-9; Two-way ANOVA with Tukey’s Multiple Comparisons Test. B) Lipid hydroperoxide levels were tested within the cardiac tissue of mice sacrificed at 6 hours following the surfactant and OLTT challenge. E2-treated males showed a trend toward increased cardiac MDA levels versus placebo males. n=3; One-way ANOVA with Bonferroni’s Multiple Comparison Test. C) E2 analysis from plasma samples taken at study termination reveal increased E2 in E2-treated males versus placebo males. n=11-13; One-way ANOVA with Bonferroni’s Multiple Comparisons Test. D) Aortic root flow cytometric gating strategy. E) Percent of aortic root macrophages positive for IsoLG. E2-treated males had higher frequency compared to placebo males. n=3; Kruskal-Wallis Test with Dunn’s Test for Multiple Comparisons.


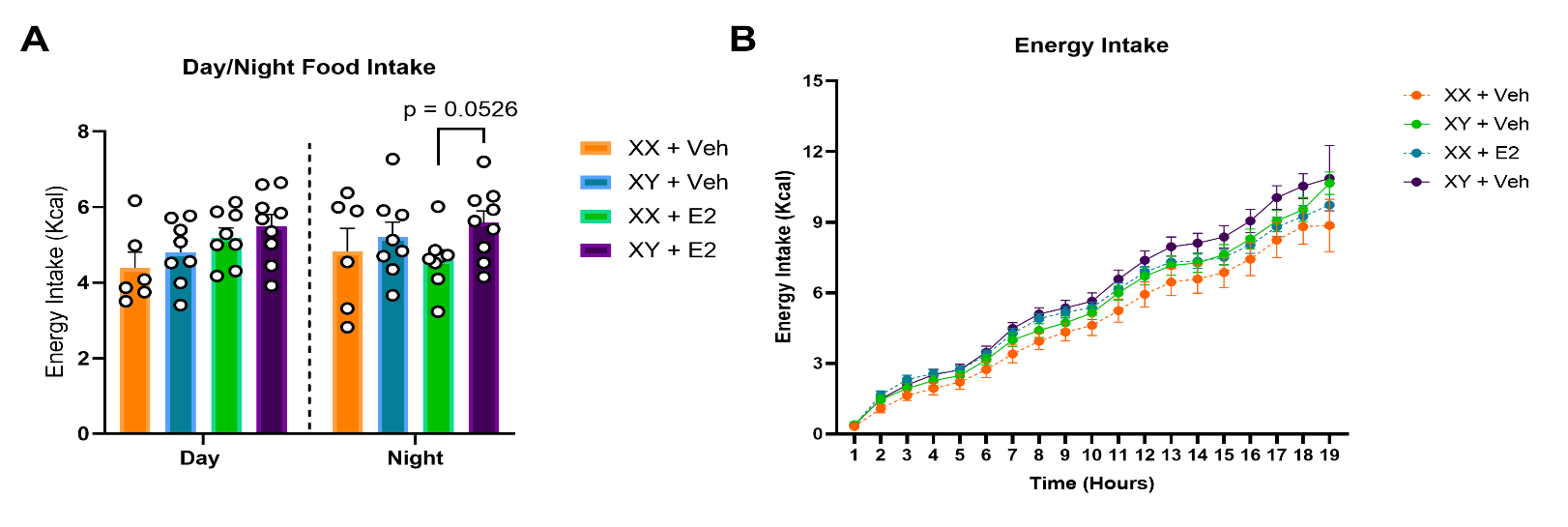


Supplemental 4. Energy intake does not differ significantly between groups but may trend higher in males during the night. A) Indirect calorimetry data of C57Bl6 mice was collected during daytime (07:00-19:00) and nighttime (19:05-06:55) at 5-minute intervals. Energy intake was summed during each cycle for each mouse. E2-treated males exhibited a trend toward increased energy intake during the night cycle. n=6-8. Two-way ANOVA with Tukey’s Multiple Comparisons Test.


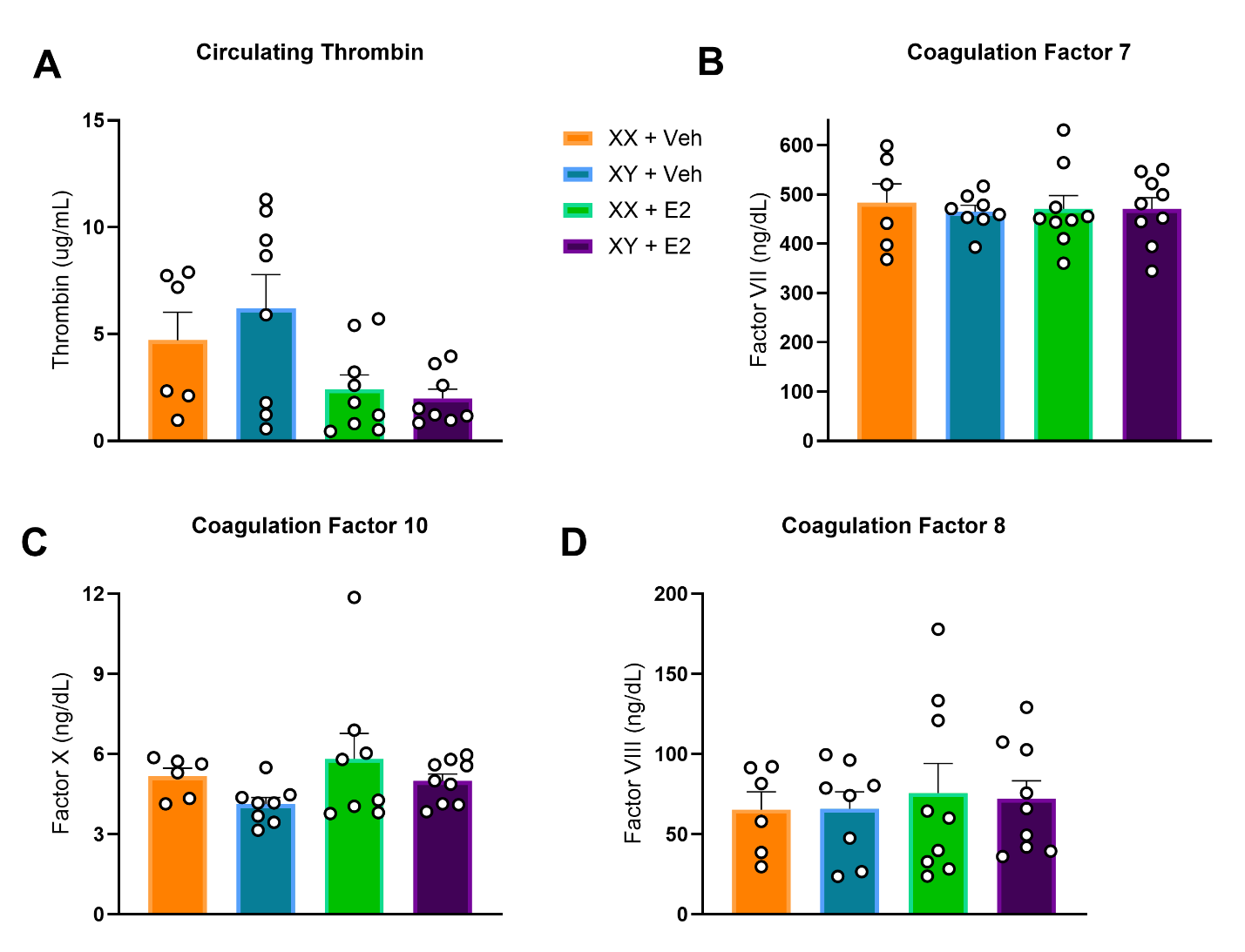


Supplemental Figure 5. E2-treatment did not significantly alter coagulation factor levels in serum. A) Thrombin ELISA results revealed no significant difference in circulating thrombin levels between groups. n=6-9; Kruskal-Wallis Test with Dunn’s Test for Multiple Comparisons. B) Coagulation factor 7 ELISA revealed no change to circulating factor 7 levels. n=6-8; One-way ANOVA with Bonferroni’s Multiple Comparison Test. C) Coagulation factor 10 ELISA showed no differences in levels between groups. n=6-9; Kruskal-Wallis Test with Dunn’s Test for Multiple Comparisons. D) Coagulation factor 8 levels were not changed between groups. n=6-8; One-way ANOVA with Bonferroni’s Multiple Comparison Test.


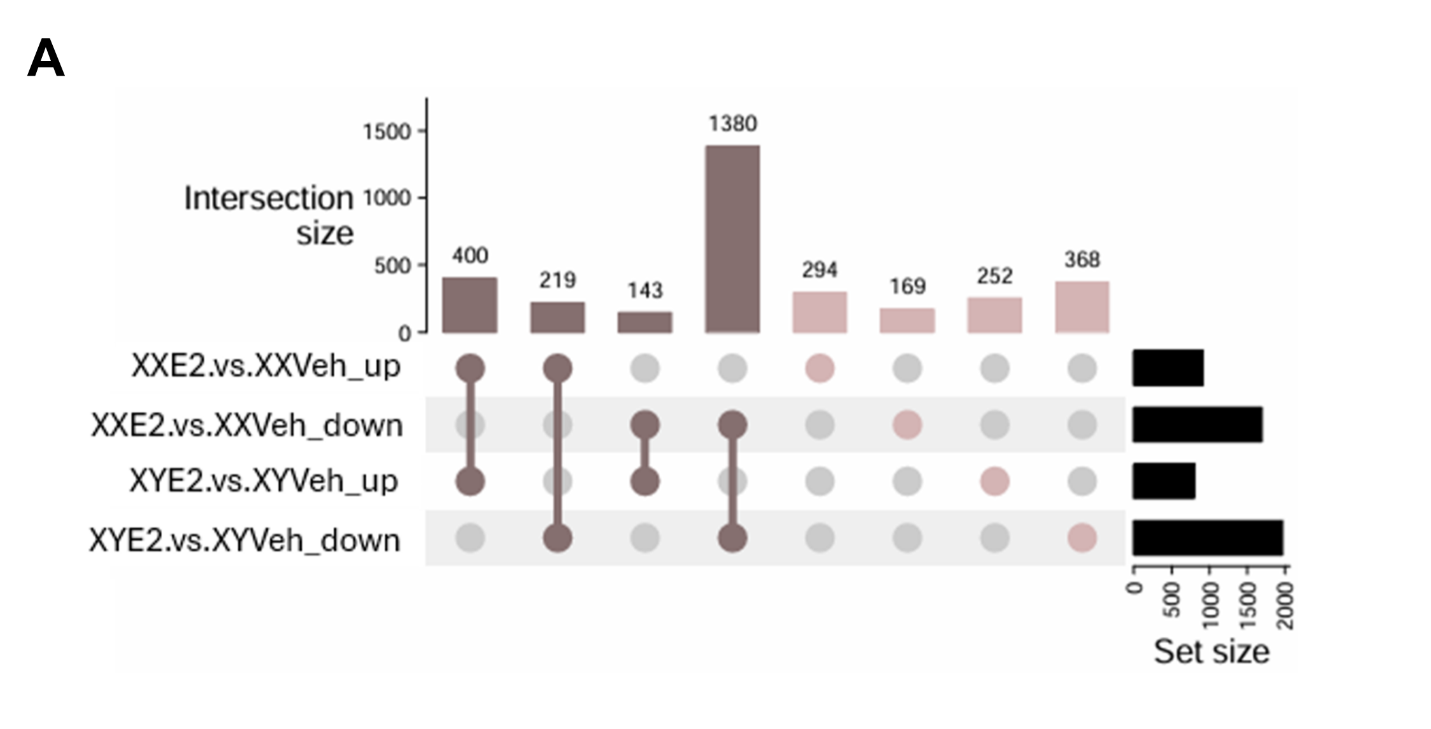


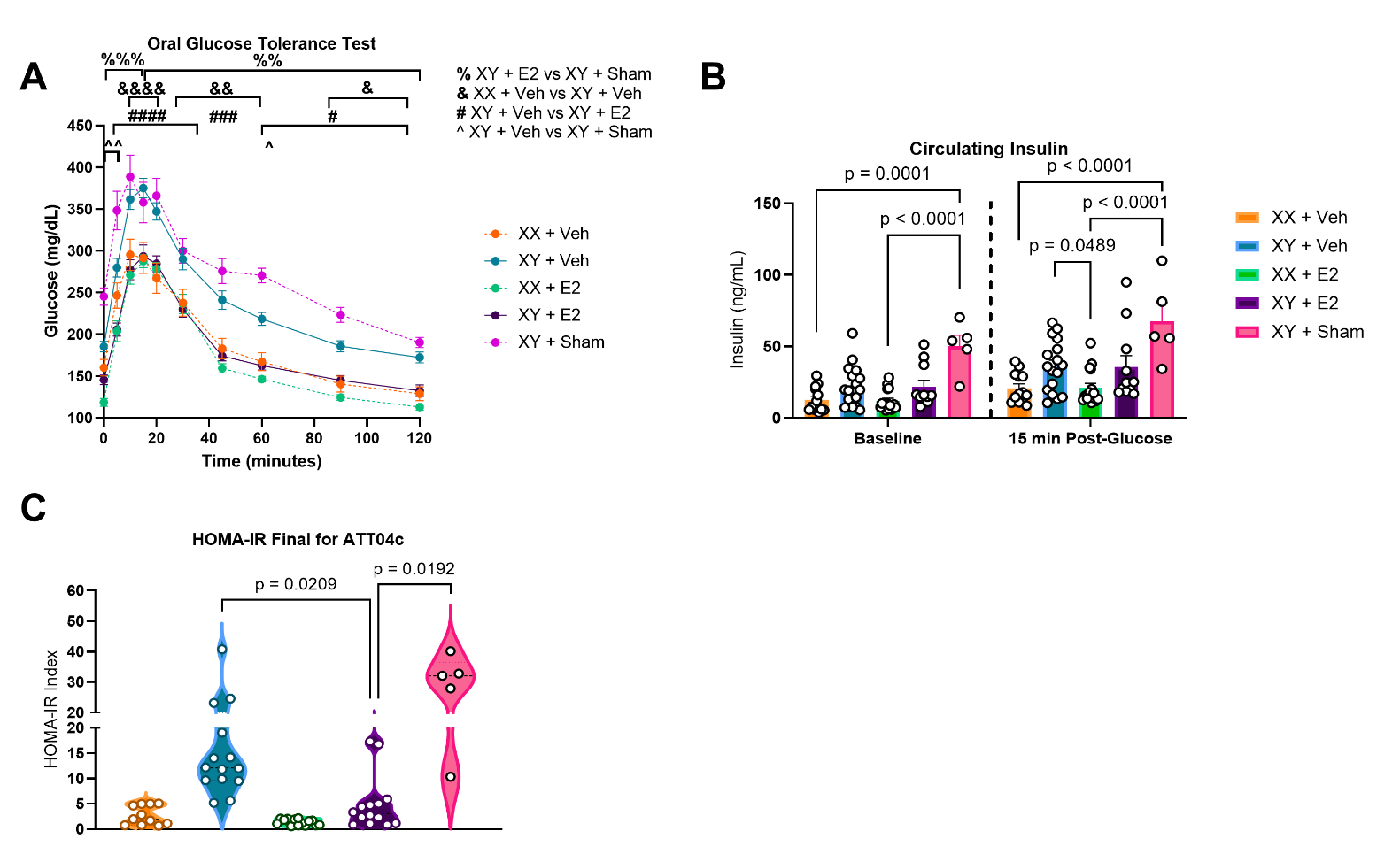


Supplemental Figure 7. Sham-operated XY males exhibited insulin resistance similar to placebo males. A) Oral glucose tolerance test was performed on 5-hour fasted mice via oral gavage. XY + Sham mice had higher plasma glucose levels when compared to placebo males at 5 and 10 minutes post-OGTT and at 90 minutes post OGTT. Sham males glucose levels were significantly higher than placebo males at 0, 5, and 90 minutes. Sham males glucose levels were significantly higher compared to E2-treated males glucose at every time point. n=5-16. (Sham males had n=5, all other groups were n=14-16). Two-way ANOVA with Tukey’s Multiple Comparisons Test. B) Tail vein blood was collected at baseline and 15 minutes post-OGTT. Sham males had higher insulin levels than both E2-treated and placebo males. E2-treated males had significantly higher insulin levels than E2-treated females at 15 minutes post-OGTT. n=5-16; Two-way ANOVA with Tukey’s Multiple Comparisons Test. C) HOMA-IR was calculated using fasting insulin and glucose. Placebo males exhibited similar HOMA-IR to sham males and both placebo and sham males had significantly increased HOMA-IR than E2-treated males. n=5-16; Kruskal-Wallis Test with Dunn’s Test for Multiple Comparisons. All mice were C57Bl/6.
